# Preparation of a furfural-derived enantioenriched vinyloxazoline building block and exploring its reactivity

**DOI:** 10.3762/bjoc.21.136

**Published:** 2025-08-29

**Authors:** Madara Darzina, Anna Lielpetere, Aigars Jirgensons

**Affiliations:** 1 Latvian Institute of Organic Synthesis Aizkraukles 21, Riga, LV-1006, Latvia; 2 Faculty of Natural Sciences and Technology, Riga Technical University, 3 P. Valdena Street, Riga LV-1048, Latvia

**Keywords:** aza-Diels–Alder reaction, electrosynthesis, furfural, valinol, vinyloxazoline

## Abstract

*N-*Alloc-protected furfuryl amino alcohols derived from furfural and ʟ- or ᴅ-valinol were subjected to Torii-type ester electrosynthesis to obtain the corresponding unsaturated esters. These served as key intermediates to prepare (*S*)- and *(R)-*enantioenriched unsaturated amides by *N*-Alloc deprotection which induced concomitant methoxymethyl group cleavage, *O-*to-*N* rearrangement, and isomerization of the double bond. An oxazoline ring formation in the resulting unsaturated amides provided the corresponding enantioenriched vinyloxazoline. The reactivity of the electron-deficient double bond in the vinyloxazoline was explored in several reactions. Out of these, the aza-Diels–Alder reaction with TsNCO was successful, leading to a highly diastereoselective formation of an oxazolo[3,2-*c*]pyrimidine derivative.

## Introduction

**Scheme 1:**
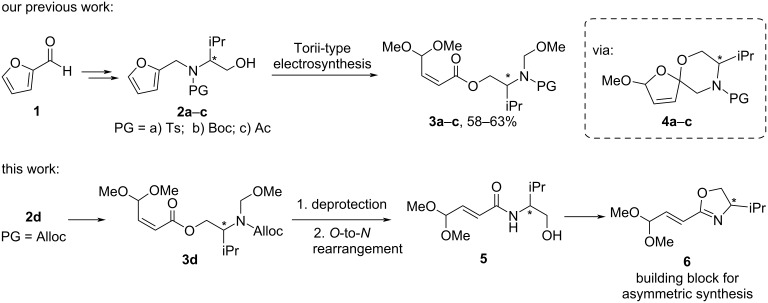
Proposed approach for the preparation of vinyloxazoline **6**.

The utilization of biomass as an alternative to fossil feedstock is central to circular economy for the production of value-added products [1–7]. Furfural [furan-2-carbaldehyde (**1**)], a platform compound derived from lignocellulosic biomass, has been utilized to obtain a range of versatile chemicals with applications to functional materials, pharmaceutically relevant compounds, and agrochemicals [8–17]. In our recent work, we have developed a Torii-type electrosynthesis of unsaturated esters **3a**–**c** starting from furfural (**1**) and amino alcohol conjugates **2a**–**c** [18]. The process involves an electrooxidative dialkoxylation of the furan ring providing spirocycles **4a**–**c** which undergo a further electrooxidative fragmentation to products **3a**–**c**. However, the further transformation of products **3a**–**c** was hampered by the problematic removal of the protecting groups (Ts, Boc, Ac) under the conditions compatible with the double bond and acetal functions. In this work, we present Torii-type electrosynthesis of ester **3d** (PG = Alloc) and its transformation to the enantioenriched vinyloxazoline building block **6**, which can be used for the asymmetric synthesis of complex molecules [19–24] (Scheme 1).

The proposed strategy relied on the *N*-deprotection of the intermediate ester **3d** inducing *O*-to-*N* rearrangement to form amide **5** as a precursor of vinyloxazoline **6**. For this purpose, Alloc (allyloxycarbonyl) turned out to be a suitable *N*-protecting group as it was compatible with the electrolysis conditions and its removal was compatible with the double bond and acetal functions in ester **3d** (see Supporting Information File 1 for attempts of other protecting groups such as Boc, Troc, and tfa). However, Pd-catalyzed *N*-Alloc deprotection induced isomerization of the double bond leading to the *trans-*isomer of amide **5**.

## Results and Discussion

**Scheme 2:**
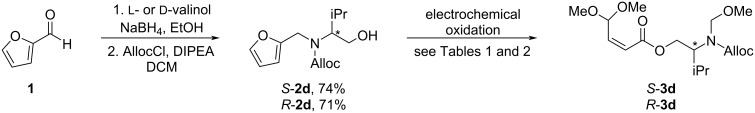
Synthesis of furfuryl amino alcohols *S-***2d** and *R-***2d** and their electrochemical oxidation to esters *S-***3d** and *R-***3d**.

The protected furfuryl amino alcohols *S*-**2d** and *R*-**2d** were prepared by reductive amination of furfural (**1**) with ʟ- and ᴅ-valinol followed by *N*-protection with Alloc-Cl (Scheme 2). The amino alcohols *S-***2d** and *R*-**2d** were then subjected to electrochemical oxidation in methanol in batch electrolysis conditions, providing unsaturated esters *S-***3d** and *R*-**3d**, respectively (Scheme 2). The previously used one-reactor two-step conditions were found to be productive for the electrosynthesis of *S-***3d**, requiring the addition of acetic acid for the intermediate spiroketal **4d** oxidation (Table 1, entry 1). We found that substrate *S-***2d** can be transformed to ester *S-***3d** in a single step with a reduced amount of HFIP as the only additive (Table 1, entries 2 and 3). Given the high cost of HFIP, we examined if it could be replaced with other proton donors for the cathode reaction, and we found that acetic acid served well for this purpose (Table 1, entry 4). Using the reaction conditions with acetic acid as additive, the reaction could be performed also in a 0.5 g scale for the synthesis of both ester enantiomers *S-***3d** and *R*-**3d** (Table 1, entries 5 and 6). In the absence of acetic acid or HFIP the electrochemical oxidation also provided the desired product **3d**, although in reduced yield (Table 1, entry 7). An increased amount of acetic acid (1 mL or ≈ 18 equiv) also reduced the yield (Table 1, entry 8). The attempt to use LiOAc as electrolyte instead of LiClO_4_ was not successful as the reaction was stopped at the spirocycle **4d** formation stage (Table 1, entry 9).

**Table 1:** Electrochemical oxidation of protected amino alcohol **2d** to ester **3d**.

Entry	Conditions^a^	Yield of **3d**

1^b^	1st step: MeOH/HFIP (10:4 mL), LiClO_4_ (1 equiv), 2.0 F; 2nd step: add AcOH (4 equiv), 3.5 F	72% (*S*-**3d**)
2	single step: MeOH/HFIP (13:1 mL), LiClO_4_ (1 equiv), 5.5 F	71% (*S*-**3d**)
3	single step: MeOH/HFIP (13.5:0.5 mL), LiClO_4_ (1 equiv), 5.5 F	70% (*S*-**3d**)
4	single step: MeOH (14 mL), AcOH (3 equiv), LiClO_4_ (1 equiv), 5.5 F	72% (*S*-**3d**)
5^b^	single step: MeOH (14 mL), AcOH (3 equiv), LiClO_4_ (0.5 equiv), 5.5 F	68%^c^ (*S*-**3d**)
6^b^	single step: MeOH (14 mL), AcOH (3 equiv), LiClO_4_ (0.5 equiv), 5.5 F	68% (*R*-**3d**)
7	single step: MeOH (14 mL), LiClO_4_ (1 equiv), 5.5 F	60% (*S*-**3d**)
8	single step: MeOH/AcOH (13:1 mL), LiClO_4_ (1 equiv), 5.5 F	55% (*S*-**3d**)
9	single step: MeOH (14 mL), AcOH (3 equiv), LiOAc (1 equiv), 5.5 F	0%^d^ (*S*-**3d**)

^a^Scale: 1 mmol. ^b^Scale: 2 mmol. ^c^Faradaic efficiency 49.5%; cell productivity 0.09 mmol/h. ^d^Major product is spirocycle **4d** (PG = Alloc).

**Scheme 3:**
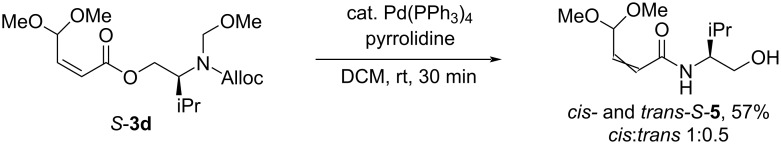
Cleavage of the *N*-Alloc group leading to a mixture of isomers *cis-S*-**5** and *trans-S-***5**.

**Scheme 4:**
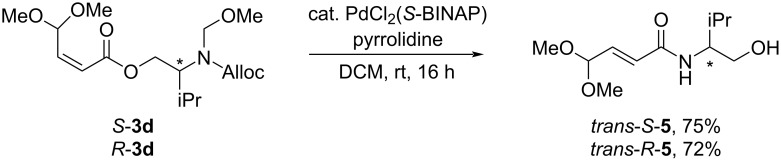
Cleavage of the *N*-Alloc group with PdCl_2_(*S*-BINAP) leading to *trans-S*-**5** and *trans-R-***5**.

The removal of the *N*-Alloc group in unsaturated ester *S-***3d** was performed using a Pd catalyst and pyrrolidine as a nucleophile. The use of Pd(PPh_3_)_4_ as the catalyst led to a fast consumption of the starting material *S-***3d** but provided a mixture of *cis-* and *trans*-amides *cis-S-***5** and *trans-S-***5** (Scheme 3). The use of PdCl_2_(*S*-BINAP) complex as a precatalyst resulted in a longer reaction time and an exclusive formation of amide *trans-S-***5** with isomerized double bond (Scheme 4). The amide *trans-R-***5** was prepared analogously from ester *R-***3d**.

Thus, Alloc was validated as non-expensive and relatively small *N-*protecting group, removal of which is compatible with double bond and acetal function of amides *S-***5** and *R-***5**. The removal of the Pd catalyst at laboratory scale was done by chromatography. For large scale synthesis, Pd scavengers have to be considered at the work-up.

**Scheme 5:**
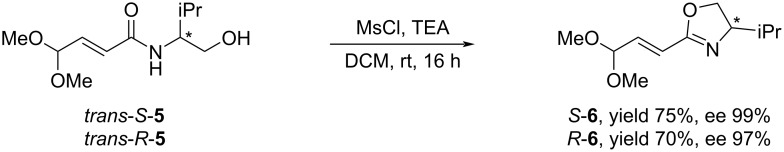
Cyclization of amides *trans*-*S-***5** and *trans*-*R-***5** to oxazolines *S-***6** and *R-***6**.

Unsaturated amides *trans-S*-**5** and *trans-R*-**5** were transformed to oxazolines *S-***6** and *R-***6** in good yields by mesylation of the hydroxy group (Scheme 5). Having both enantiomers in hand, the enantiomeric excess of oxazolines *S-***6** and *R-***6** was determined by chiral HPLC. This confirmed that no erosion of enantiomeric purity had happened during the deprotection stage.

**Scheme 6:**
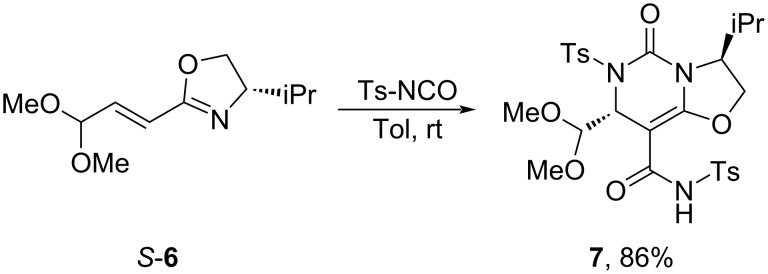
aza-Diels–Alder reaction of vinyloxazoline *S-***6** with TsNCO.

With the enantioenriched vinyloxazoline *S-***6** in hand, we explored the reaction scope involving its electron-deficient double bond. Unfortunately, the olefin appeared unreactive or gave a mixture of products in copper-catalyzed 1,4-addition of phenylmagnesium bromide, Giese reaction with 2-iodopropane, Simmons‒Smith or Johnson–Corey–Chaykovsky cyclopropanation, hydroboration reaction with 9-BBN, and Diels–Alders reaction with Danishevsky diene. Gratifyingly, it was found that vinyloxazoline *S-***6** is a good substrate for an aza-Diels–Alder reaction with tosylisocyanate (TsNCO) providing the oxazolo[3,2-*c*]pyrimidine derivative **7** as the only detectable diastereomer (Scheme 6) [22–24]. Oxazolo[3,2-*c*]pyrimidines are substructures in several pharmaceutically relevant compounds such as potent gonadotropin-releasing hormone receptor antagonists with potential application as anticancer drugs [25] and as nucleoside analogs with antiviral potency [26].

**Scheme 7:**
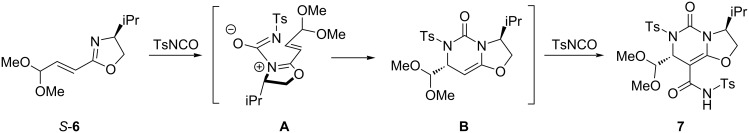
The proposed mechanism of product **7** formation.

According to the reaction mechanism proposed by Elliott et al., the aza-Diels–Alder reaction of vinyloxazoline *S-***6** with TsNCO is a step-wise process [22]. The first step involves addition of the oxazoline nitrogen to TsNCO leading to a zwitterionic intermediate **A**, which undergoes 1,4-conjugate addition forming a cyclic intermediate **B**. Subsequently, the electron-rich double bond in intermediate **B** reacts with a second equivalent of TsNCO to form oxazolo[3,2-*c*]pyrimidine derivative **7** (Scheme 7).

The highly diastereoselective formation of product **7** can be explained based on the theoretical calculations by Elliott et al. of aza-Diels–Alder reaction of vinyloxazoline with TsNCO [19]. According to their investigations, the aza-nucleophile attack to the double bond in intermediate **A** is kinetically preferred from the face forming the *R*-configured carbon of the C–N bond as a result of a minimized steric interaction of the oxygen in zwitterion with iPr substituent of oxazoline.

## Conclusion

Unsaturated ester obtained by Torii-type ester electrosynthesis from the conjugate of two biobased starting materials furfural and valinol serves as an intermediate to prepare an enantioenriched vinyl oxazoline **6** with atom economy 24.5% over 5 steps (>75% average per step). The electrochemical oxidation of *N-*Alloc can be performed as a single step operation in MeOH with acetic acid as an additive. This is a step toward the large scale synthesis of enantioenriched vinyloxazolines **6** from biomass-derived furfural, however, several challenges remain such as replacing LiClO_4_ as electrolyte at the oxidation step and avoiding chromatography for purification. Further scale-up could be achieved by switching to flow conditions, or designing a continuous tank reactor [27].

Several attempts were made to explore enantioenriched vinyloxazoline **6** as a chiral building block. While additions to the double bond were not successful, vinyloxazoline **6** was found to be a competent substrate for the aza-Diels–Alder reaction in with TsNCO to give oxazolo[3,2-*c*]pyrimidine derivative **7** as a single diastereomer.

## Supporting Information

File 1 Experimental procedures, characterization data and copies of NMR spectra.

## Data Availability

All data that supports the findings of this study is available in the published article and/or the supporting information of this article.
