# Supplementary material for: Preparation of a furfural-derived enantioenriched vinyloxazoline building block and exploring its reactivity
Source: Beilstein J Org Chem. 2025 Aug 29;21:1737–41. doi: 10.3762/bjoc.21.136 (PMC12415904; doi:10.3762/bjoc.21.136)

BEILSTEIN JOURNAL OF ORGANIC CHEMISTRY

# Supporting Information

for

# Preparation of a furfural-derived enantioenriched vinyloxazoline building block and exploring its reactivity

Madara Darzina, Anna Lielpetere and Aigars Jirgensons

*Beilstein J. Org. Chem.* **2025,** *21,* 1737–1741. doi:10.3762/bjoc.21.136

# Experimental procedures, characterization data and copies of NMR spectra

License and Terms: This is a supporting information file under the terms of the Creative Commons Attribution License (https://creativecommons.org/licenses/by/4.0). Please note that the reuse, redistribution and reproduction in particular requires that the author(s) and source are credited and that individual graphics may be subject to special legal provisions.
The license is subject to the *Beilstein Journal of Organic Chemistry* terms and conditions: (https://www.beilstein-journals.org/bjoc/terms)

## Table of contents


General considerations ........ S2

Selection of the protecting group for the vinyloxazoline **6** synthesis........ S4

Synthesis of Alloc-protected amino alcohol **2d** ........ S5

Electrosynthesis of unsaturated ester **3d** ........ S7

Synthesis of unsaturated amide **5** ........ S9

Synthesis of vinyl oxazoline **6**........ S11

Synthesis of oxazolo[3,2-*c*]pyrimidine **7** ........ S12

Chiral HPLC analysis of *rac*-**6** ........ S13

Chiral HPLC analysis of *S*-**6** ........ S14

Chiral HPLC analysis of *R*-**6** ........ S15

Copies of NMR spectra ........ S16

**Figure S1**. $^1$H NMR of compound *S*-**S1** ($CDCl_3$, 300 MHz) ........ S17

**Figure S2**. $^1$H NMR of compound *S*-**2d** (DMSO-$d_6$, 400 MHz)........ S18

**Figure S3**. $^{13}C\{^1H\}$ NMR of compound *S*-**2d** (DMSO-$d_6$, 101 MHz)........ S19

**Figure S4**. $^1$H NMR of compound *S*-**3d** ($CD_3CN$, 400 MHz) ........ S20

**Figure S5**. $^{13}C\{^1H\}$ NMR of compound *S*-**3d** ($CD_3CN$, 101 MHz). ........ S21

**Figure S6**. $^1$H NMR of compound *trans*-*S*-**5** ($CDCl_3$, 400 MHz)........ S22

**Figure S7**. $^{13}C\{^1H\}$ NMR of compound *trans*-*S*-**5** ($CDCl_3$, 101 MHz). ........ S23

**Figure S8**. $^1$H NMR of compound *cis*-*S*-**5** ($CDCl_3$, 400 MHz)........ S24

**Figure S9**. $^{13}C\{^1H\}$ NMR of compound *cis*-*S*-**5** ($CDCl_3$, 101 MHz)........ S25

**Figure S10**. $^1$H NMR of compound *S*-**6** ($CDCl_3$, 400 MHz) ........ S26

**Figure S11**. $^{13}C\{^1H\}$ NMR of compound *S*-**6** ($CDCl_3$, 101 MHz). ........ S27

**Figure S12**. $^1$H NMR of compound **7** ($CD_3CN$, 400 MHz). ........ S28

**Figure S13**. $^{13}C\{^1H\}$ NMR of compound **7** ($CD_3CN$, 101 MHz). ........ S29

# General considerations

Reagents and starting materials for synthesis were obtained from commercial sources and used as received. All solvents were used as received. For electrolysis, MeOH (≥99.8%, HPLC grade) from Fisher Chemical and HFIP (>99.5%) from Fluorochem were used.

$^{1}H$ NMR, $^{13}C$ NMR, and 2D spectra were recorded on 400 MHz Bruker Avance Neo and 300 MHz Bruker UltraShield spectrometers. Chemical shift values (δ) are given in parts per million (ppm), using residual solvent as an internal standard. Coupling constants are reported as *J* values in hertz (Hz). Structural assignments were made with additional information from COSY, NOESY, HSQC, and HMBC experiments.

High resolution mass spectra (HRMS) were recorded on a Waters Synapt G2-Si TOF MS instrument in ESI technique.

Specific optical rotations were measured at 20 °C on a Rudolph Research Analytical Autopol VI polarimeter, cell length 100 mm at 589 nm. $CHCl_3$ was used as solvent and concentration is specified for each sample.

Flash column chromatography was carried out using silica gel (230–400 mesh). Thin-layer chromatography (TLC) was performed on silica gel and was visualized by a UV lamp or staining with potassium permanganate. Reversed-phase flash chromatography was carried out on a Biotage Isolera One flash chromatograph, using commercially available C18 silica gel columns.

Batch electrolysis was performed using a potentiostat ElectraSyn 2.0 in constant current conditions, using undivided 20 mL ElectraSyn cells. Graphite (SK-50; dimensions (W × H × D): 8 × 52.5 × 2 mm) electrodes from IKA were used. The distance between electrodes is 0.8 cm; during electrolysis 2.4 cm of the electrode was submerged in solution. Graphite electrodes were polished with emery paper before each experiment. During electrolysis, the reaction mixture was stirred at 750 rpm. Electrolysis was performed at room temperature (≈20 °C) and under atmospheric conditions.

**Photo of batch electrolysis set-up:**

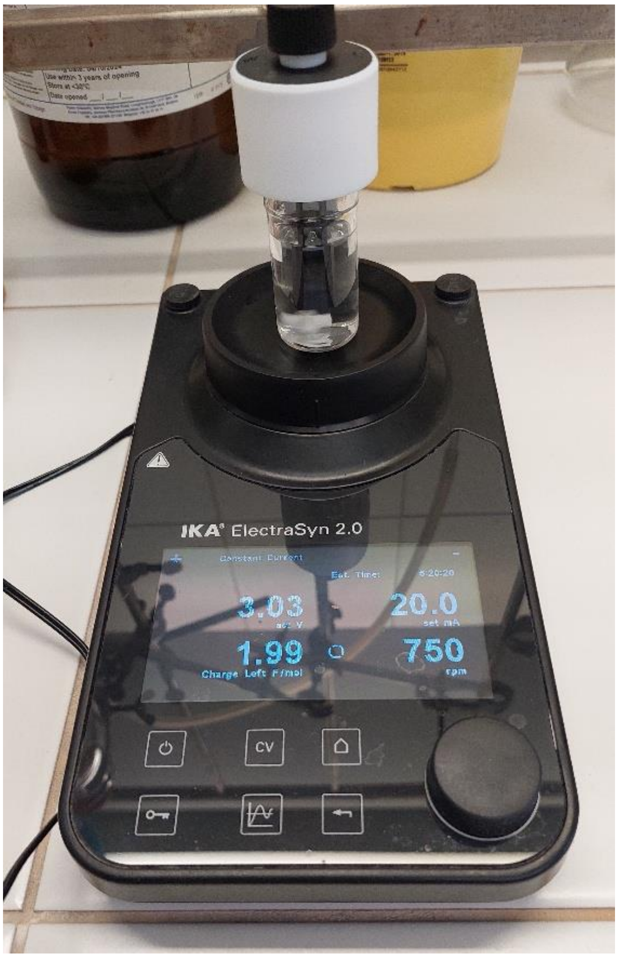

## Selection of the protecting group for the vinyloxazoline 6 synthesis

Attempts to deprotect the Boc group in ester **2d** were not successful (Scheme 1), likely due to reactivity of acetal group in strongly acidic conditions.

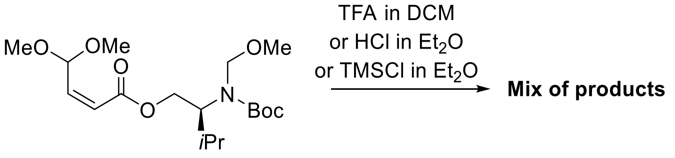


**2b** (prepared as described in ref. 1)

**Scheme S1.** Attempt to deprotect ester **2d**.

Therefore, alternative protecting groups such as Alloc, Troc and tfa were explored. The corresponding protected amino alcohols *S*-**2d**, **S2**, **S3** were prepared from intermediate *S*-**S1**. Oxidation of the protected amino alcohols *S*-**2d**, **S2**, **S3** was performed by a one-pot two-step procedure, using the previously established conditions for Torii-type ester synthesis (ref. 1). Only the Alloc-protected substrate *S*-**2d** provided the expected ester *S*-**3d**.

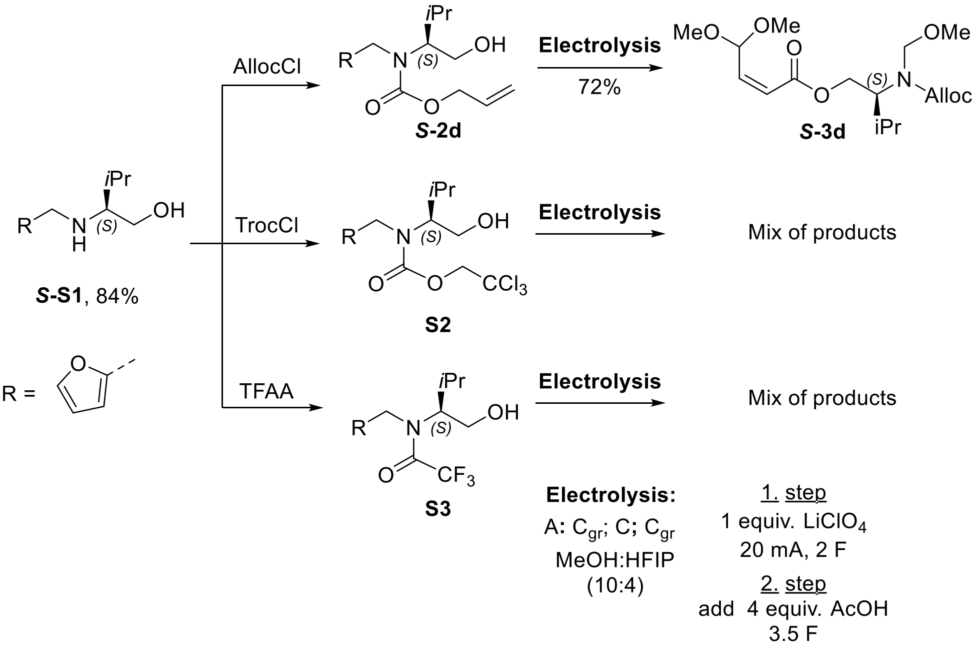


**Scheme S2.** Synthesis of *N*-protected amino alcohols *S*-**2d**, **S2, S3** and their electrochemical oxidation.

[1]: Dārziņa, M.; Lielpētere, A.; Jirgensons, A. Torii-type electrosynthesis of α,β-unsaturated esters from furfurylated ethylene glycols and amino alcohols. *Eur. J. Org. Chem.* **2021**, 4224–4228. https://doi.org/10.1002/ejoc.202100605

## Synthesis of Alloc-protected amino alcohol 2d

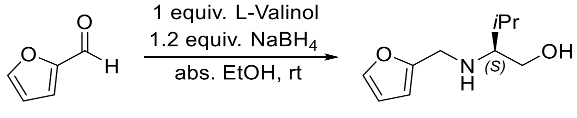


**1** **S-S1**, 84%

The synthesis was performed according to a known literature procedure[1]. Furfural (**1**, 2.00 mL; 1.0 equiv; 24.1 mmol) and L-valinol ((*S*)-valinol, 2.49 g; 1.0 equiv; 24.1 mmol) were dissolved in abs. EtOH (20 mL) under argon atmosphere and left to stir at room temperature overnight. After 16 h, the reaction mixture was cooled in an ice bath and $NaBH_4$ (1.10 g; 1.2 equiv; 29.0 mmol) was added in small portions. Then, the reaction mixture was left to stir for another 20 h at room temperature. Afterwards, the reaction mixture was cooled in an ice bath and aqueous 5% $KHSO_4$ solution was added slowly until gas evolution ceased. Ethanol was evaporated and the reaction mixture was partitioned between $Et_2O$ and aqueous 5% $KHSO_4$ solution. The organic layer was washed with $KHSO_4$ solution four more times. The aqueous layers were combined and carefully neutralized by addition of solid $NaHCO_3$ until pH was >7. The aqueous phase was washed with a $CHCl_3$/iPrOH 3:1 mixture four times, the organic layers were combined, dried with $Na_2SO_4$, filtered and the solvent was evaporated. Product *S*-**S1** (3.73 g; 84%) was obtained as an orange oil. The product was used in the next step without further purification. The $^1$H NMR spectrum was in agreement with that reported in the literature[1].

**$^1$H NMR** (300 MHz, $CDCl_3$, ppm) δ 7.37 (dd, *J* = 1.9, 0.8 Hz, 1H), 6.32 (dd, *J* = 3.2, 1.9 Hz, 1H), 6.22 (dd, *J* = 3.2, 0.7 Hz, 1H), 3.95 – 3.78 (m, 2H), 3.65 (dd, *J* = 11.0, 4.1 Hz, 1H), 3.43 (dd, *J* = 11.0, 6.8 Hz, 1H), 2.79 (s, 3H), 2.48 (td, *J* = 6.6, 4.1 Hz, 1H), 1.83 (dq, *J* = 13.5, 6.8 Hz, 1H), 0.98 – 0.88 (m, 6H).

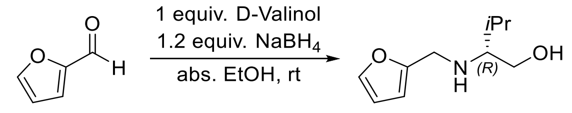


**1** **R-S1**, 88%

Synthesis of *R*-**S1** was performed using the same procedure as for *S*-**S1**. Furfural (**1**, 2.04 mL; 1.0 equiv; 24.6 mmol), D-valinol ((*R*)-valinol, 2.54 g; 1.0 equiv; 24.6 mmol) and $NaBH_4$ (1.11 g; 1.2 equiv; 29.5 mmol) were used. Product *R*-**S1** (3.96 g; 88%) was obtained as an orange oil. The $^1$H NMR spectrum was in agreement with that reported in the literature[1].

**$^1$H NMR** spectrum for *R*-**S1** matches that of *S*-**S1**.

[1]: Dārziņa, M.; Lielpētere, A.; Jirgensons, A. Torii-type electrosynthesis of α,β-unsaturated esters from furfurylated ethylene glycols and amino alcohols. *Eur. J. Org. Chem.* **2021**, 4224–4228. https://doi.org/10.1002/ejoc.202100605

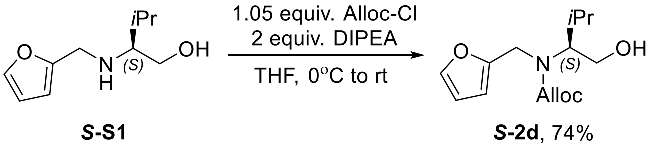


Substrate *S*-**S1** (1.43 g; 1.0 equiv; 7.80 mmol) was dissolved in dry THF (10 mL) under argon atmosphere. The flask was cooled in an ice bath and DIPEA (2.70 mL; 2.0 equiv; 15.6 mmol) was added to the reaction mixture. Then, allyl chloroformate (0.87 mL; 1.05 equiv; 8.19 mmol) was added dropwise. The mixture was left to stir at 0 °C for 2 hours, then allowed to warm to room temperature and stirred overnight. The solvent was evaporated and the mixture was dissolved in EtOAc (20 mL) and transferred into a separatory funnel. The organic layer was washed with sat. $NH_4Cl$ solution (1 ×), sat. $NaHCO_3$ solution (1 ×) and sat. NaCl solution (1 ×). Then, the organic layer was dried with $Na_2SO_4$, filtered and the solvent was evaporated. The crude product was then purified using flash chromatography system on reversed-phase using C18 silica gel column. Eluent system $H_2O$/MeCN, gradient 10% to 100% MeCN. Product *S*-**2d** (1.55 g; 74%) was obtained as a colorless oil.

**Note: Compound observed as rotamers.**

**$^1$H NMR** (400 MHz, DMSO-$d_6$, ppm) δ 7.50 (dd, *J* = 1.9, 0.9 Hz, 1H), 6.36 (dd, *J* = 3.2, 1.8 Hz, 1H), 6.29 (dd, *J* = 3.1, 0.6 Hz, 1H), 5.91 (ddt, *J* = 17.3, 10.5, 5.2 Hz, 1H), 5.28 (dq, *J* = 17.3, 1.7 Hz, 1H), 5.17 (dq, *J* = 10.6, 1.5 Hz, 1H), 4.54 (dq, *J* = 5.3, 1.5 Hz, 2H), 4.50 – 4.31 (m, 3H), 3.63 (t, *J* = 5.5 Hz, 2H), 3.49 (dt, *J* = 9.9, 6.1 Hz, 1H), 1.90 (s, 1H), 0.89 (d, *J* = 6.6 Hz, 3H), 0.66 (d, *J* = 6.7 Hz, 3H). **Note: spectra taken at 75 °C**.

**$^{13}$C{$^1$H} NMR** (101 MHz, DMSO-$d_6$, ppm) δ 155.4 (broad signal), 152.2, 141.4, 133.3, 116.3, 110.2, 107.5, 65.0, 64.8, 60.4, 41.5, 27.4 (broad signal), 19.9, 19.5. **Note: spectra taken at 60 °C**.

**HRMS** (ESI) m/z: $[M+Na]^+$ Calcd for $C_{14}H_{21}NO_4Na$ 290.1363; Found 290.1372.

*S*-**2d**: $\alpha_D^{20} + 8.6\ (c\ 1.16; \mathrm{CHCl_3})$.

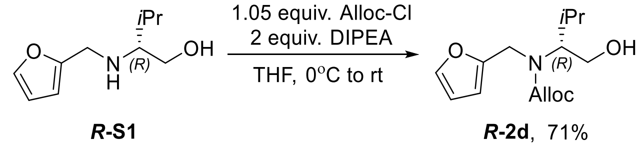


The synthesis of *R*-**2d** was performed using the same procedure as for *S*-**2d**. Substrate *R*-**S1** (2.01 g; 1.0 equiv; 11.0 mmol), DIPEA (3.82 mL; 2.0 equiv; 21.9 mmol) and allyl chloroformate (1.22 mL; 1.05 equiv; 11.5 mmol) were used. Product *R*-**2d** (2.08 g; 71%) was obtained as a colorless oil.

**$^1$H NMR** and **$^{13}$C NMR** spectra for *R*-**2d** matches that of *S*-**2d**.

*R*-**2d**: $\alpha_D^{20} - 8.6\ (c\ 1.02; \mathrm{CHCl_3})$.

## Electrosynthesis of unsaturated ester 3d

**<u>Two-step electrolysis of substrate *S*-2d at 2 mmol scale:</u>**

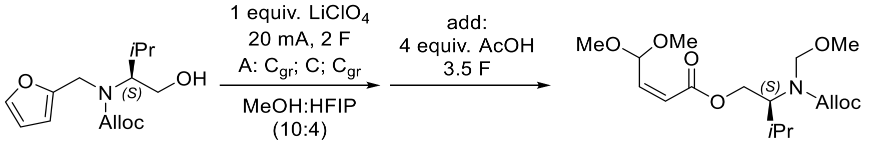


***S*-2d** ***S*-3d**, 72% (2 mmol scale)

Substrate *S*-**2d** (534 mg; 1.0 equiv; 2.00 mmol) and $LiClO_4$ (212 mg; 1.0 equiv; 2.00 mmol) were transferred into a 20 mL electrolytic cell and dissolved in 10 mL MeOH and 4 mL HFIP mixture. The electrolytic cell was fitted with a graphite anode and cathode. Constant current electrolysis was performed at 20 mA ($j$ = 10.4 mA/cm$^2$, distance between electrodes 0.8 cm), ambient conditions, until 2 F of charge was passed through the solution. Then, AcOH (0.46 mL; 4.0 equiv; 8.00 mmol) was added to the reaction mixture and electrolysis was continued at 20 mA until additional 3.5 F of charge was passed through the solution. Total electrolysis time: approx. 15 hours.

After electrolysis, TEA (1.11 mL; 4.0 equiv; 8.00 mmol) was added to the electrolytic cell and the reaction mixture was diluted with approx. 20 mL EtOAc. The solution was filtered through a pad of silica gel and the solvent was evaporated. The product was purified using column chromatography, eluent system Hex/MTBE/acetone 6:1:1, product $R_f \approx 0.4$). Product *S*-**3d** (514 mg; 72%) was obtained as a colorless oil.

**<u>Single-step electrolysis of substrate *S*-2d at 2 mmol scale:</u>**

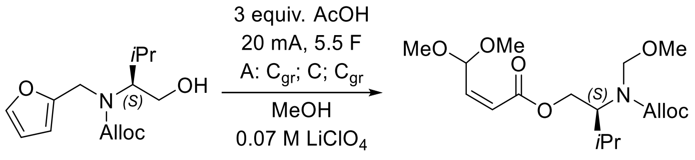


***S*-2d** ***S*-3d**, 68% (2 mmol scale)

Substrate *S*-**2d** (534 mg; 1.0 equiv; 2.00 mmol) and $LiClO_4$ (106 mg; 0.5 equiv; 1.00 mmol; 0.07 M) were transferred into a 20 mL electrolytic cell and dissolved in 14 mL MeOH. Then, AcOH (345 uL; 3.0 equiv.; 6.03 mmol) was added to the reaction mixture. The electrolytic cell was fitted with graphite anode and cathode. Constant current electrolysis was performed at 20 mA ($j$ = 10.4 mA/cm$^2$, distance between electrodes 0.8 cm), ambient conditions, until 5.5 F of charge was passed through the solution. Total electrolysis time: approx. 15 hours.

After electrolysis, TEA (2.78 mL; 10.0 equiv; 20.0 mmol) was added to the electrolytic cell and the reaction mixture was concentrated on a rotovap. Then, the reaction mixture was diluted with approx. 10 mL EtOAc and filtered through a pad of silica gel and the solvent was evaporated. The product was purified using column chromatography, eluent system Hex/MTBE/acetone 6:1:1, product $R_f \approx 0.4$). Product *S*-**3d** (485 mg; 68%) was obtained as a colorless oil.

**Note: Compound observed as rotamers.**

**$^1$H NMR** (400 MHz, $CD_3CN$, ppm) δ 6.08 (dd, *J* = 11.8, 7.5 Hz, 1H), 6.02 – 5.88 (m, 2H), 5.68 (dd, *J* = 7.5, 1.0 Hz, 1H), 5.31 (dq, *J* = 17.3, 1.7 Hz, 1H), 5.20 (dq, *J* = 10.6, 1.5 Hz, 1H), 4.74 – 4.69 (m, 1H), 4.68 – 4.55 (m, 3H), 4.39 (dd, *J* = 11.7, 4.2 Hz, 1H), 4.34 – 4.29 (m, 1H), 3.77 (td, *J* = 9.7, 3.9 Hz, 1H), 3.33 (d, *J* = 1.1 Hz, 6H), 3.25 (s, 3H), 2.05 – 2.00 (m, 1H), 1.01 (d, *J* = 6.6 Hz, 3H), 0.89 (d, *J* = 6.7 Hz, 3H). **Note: spectra taken at 40 °C**.
**$^{13}$C{$^1$H} NMR** (101 MHz, $CD_3CN$, ppm) δ 165.9, 164.7, 157.8, 156.8, 144.1, 143.8, 134.2, 122.9, 122.8, 117.7, 117.4, 99.4, 78.2, 66.8, 66.6, 65.1 64.7, 62.9, 56.5, 56.1, 54.2, 29.4, 29.0, 20.4, 20.3.
**HRMS** (ESI) m/z: $[M+Na]^+$ Calcd for $C_{17}H_{29}NO_7Na$382.1836; Found 382.1840.
*S*-**3d**: $\alpha_D^{20} + 15.9\ (c\ 0.97; \mathrm{CHCl_3})$.

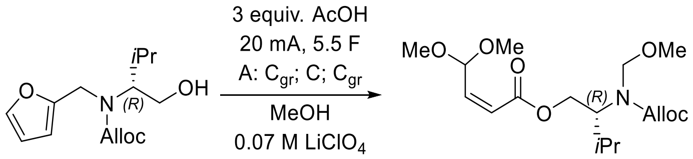


***R*-2d** ***R*-3d**, 68%

The synthesis of *R*-**3d** was performed using the same single step procedure as for *S*-**3d** (2 mmol scale). Substrate *R*-**2d** (534 mg; 1.0 equiv; 2.00 mmol), $LiClO_4$ (106 mg; 0.5 equiv; 1.00 mmol), AcOH (345 uL; 3.0 equiv; 6.03 mmol) and TEA (2.78 mL; 10.0 equiv; 20.0 mmol) were used. Product *R*-**3d** (490 mg; 68%) was obtained as a colorless oil.
**$^1$H NMR** and **$^{13}$C NMR** spectra for *R*-**3d** matches that of *S*-**3d**.
*R*-**3d**: $\alpha_D^{20} - 15.6\ (c\ 1.00; \mathrm{CHCl_3})$.

## Synthesis of unsaturated amide 5

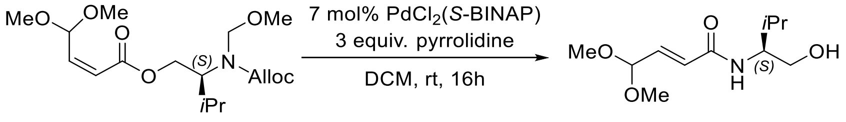


***S*-3d** ***trans*-*S*-5**, 75%

Substrate *S*-**3d** (502 mg; 1.0 equiv; 1.40 mmol) was dissolved in dry DCM (15 mL). Argon gas was slowly bubbled through the solution for 10 minutes, then catalyst $PdCl_2$(*S*-BINAP) (78.4 mg; 0.07 equiv; 0.10 mmol) was added to the solution and argon gas was bubbled through the solution for 5 more minutes. Then flask was sealed with a septum and pyrrolidine (0.34 mL; 3.0 equiv; 4.19 mmol) was added to the reaction mixture. The reaction was left to stir overnight at room temperature. After about 16 hours, the reaction mixture was transferred into a separatory funnel and partitioned between DCM and sat. aqueous $NH_4Cl$ solution. The aqueous layer was washed with $CHCl_3$/iPrOH 3:1 mixture two more times, the organic layers were combined, dried with $Na_2SO_4$, filtered and the solvent was evaporated. The product was purified using column chromatography, eluent system MTBE/acetone 3:1, product $R_f \approx 0.45$). Product *trans*-*S*-**5** (242 mg; 75%) was obtained as a while solid.

**$^1$H NMR** (400 MHz, $CDCl_3$, ppm) δ 6.67 (dd, *J* = 15.5, 3.7 Hz, 1H), 6.18 (dd, *J* = 15.5, 1.4 Hz, 1H), 6.11 (d, *J* = 8.6 Hz, 1H), 4.94 (dd, *J* = 3.8, 1.4 Hz, 1H), 3.83 – 3.75 (m, 1H), 3.73 – 3.63 (m, 2H), 3.32 (s, 6H), 3.09 (s, 1H), 1.98 – 1.84 (m, *J* = 6.8 Hz, 1H), 1.02 – 0.85 (m, 6H).

**$^{13}$C{$^1$H} NMR** (101 MHz, $CDCl_3$, ppm) δ 165.9 139.1, 127.0, 100.7, 63.6, 57.3, 52.9, 29.2, 19.9, 19.0.

**HRMS** (ESI) m/z: $[M+H]^+$ Calcd for $C_{11}H_{22}NO_4$ 232.1543; Found 232.1558.

*trans*-*S*-**5**: $\alpha_D^{20} - 41.0\ (c\ 1.07; \mathrm{CHCl_3})$.

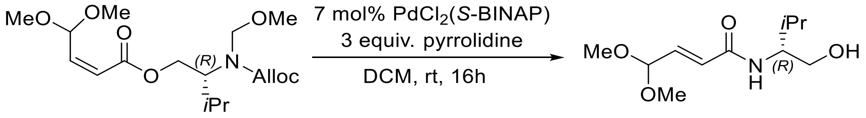


***R*-3d** ***trans*-*R*-5**, 72%

The synthesis of *trans*-*R*-**5** was performed using the same procedure as for *trans*-*S*-**5**. Substrate *R*-**3d** (363 mg; 1.0 equiv; 1.01 mmol), $PdCl_2$(*S*-BINAP) (56.7 mg; 0.07 equiv; 0.07 mmol) and pyrrolidine (0.25 mL; 3.00 equiv; 3.03 mmol) were used. The product *trans*-*R*-**5** (169 mg; 72%) was obtained as a white solid.

**$^1$H NMR** and **$^{13}$C NMR** spectra for *trans*-*R*-**5** matches that of *trans*-*S*-**5**.

*trans*-*R*-**5**: $\alpha_D^{20} + 39.1\ (c\ 1.00; \mathrm{CHCl_3})$.

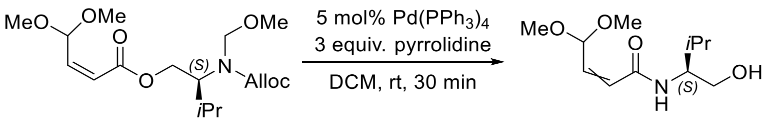


***S*-3d** **→** ***S*-5**, 57%
cis:trans (1:0.5)

Substrate *S*-**3d** (158 mg; 1.0 equiv; 0.44 mmol) and pyrrolidine (0.11 mL; 3.0 equiv; 1.32 mmol) were dissolved in dry DCM (5 mL). Argon gas was slowly bubbled through the solution for 10 minutes, then catalyst $Pd(PPh_3)_4$ (25.4 mg; 0.02 equiv; 0.05 mmol) was added to the solution and argon gas was bubbled through the solution for 2 more minutes. Then, the flask was sealed with a septum and the reaction was left to stir at room temperature. Reaction progress was monitored via TLC. When all starting material was consumed (about 30 min), the reaction mixture was transferred into a separatory funnel and partitioned between DCM and sat. aqueous $NH_4Cl$ solution. The aqueous layer was washed with DCM two more times, the organic layers were combined, dried with $Na_2SO_4$, filtered and the solvent was evaporated. At this stage, double bond isomeric ratio was determined to be 1:0.5 (*cis*:*trans*; determined by $^1$H NMR).

The products were purified using column chromatography, eluent system MTBE/acetone 3:1, *cis*-*S*-**5** $R_f$ ≈ 0.54, *trans*-*S*-**5** $R_f$ ≈ 0.45), followed by flash chromatography on reversed phase, using C18 silica gel column. Eluent system $H_2O$/MeCN, gradient 2% to 30% MeCN. Products *S*-**5** (58 mg; 57%) was obtained as a *cis*:*trans* mixture (1:0.5; determined by $^1$H NMR).

Isomer *cis*-*S*-**5** was then separated for characterization, using reversed-phase flash chromatography in the same conditions as described above.

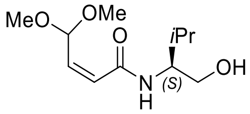


***cis*-*S*-5**

**$^1$H NMR** (400 MHz, $CDCl_3$, ppm) δ 6.71 (d, *J* = 9.1 Hz, 1H), 5.98 (dd, *J* = 12.1, 0.6 Hz, 1H), 5.92 (dd, *J* = 12.1, 5.9 Hz, 1H), 5.54 (dd, *J* = 5.9, 0.6 Hz, 1H), 3.79 (dtd, *J* = 8.3, 6.4, 3.5 Hz, 1H), 3.74 – 3.62 (m, 2H), 3.37 (d, *J* = 1.3 Hz, 6H), 1.98 – 1.85 (m, *J* = 6.8 Hz, 1H), 0.99 – 0.92 (m, 6H).
**$^{13}$C{$^1$H} NMR** (101 MHz, $CDCl_3$, ppm) δ 166.4, 137.1, 127.7, 99.0, 64.0, 57.5, 53.4, 53.2, 29.1, 19.6, 18.7.
**HRMS** (ESI) m/z: $[M+Na]^+$ Calcd for $C_{11}H_{21}NO_4Na$ 254.1362; Found 254.1366.

**Synthesis of vinyloxazoline 6**

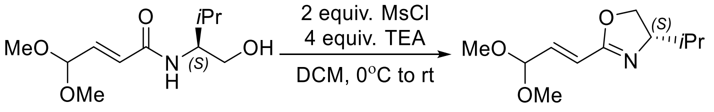


In a heat-dried flask under Ar atmosphere, substrate *trans-S*-**5** (374 mg; 1.0 equiv; 1.62 mmol) was dissolved in dry DCM (7 mL). The flask was cooled in an ice bath and TEA was added (0.90 mL; 4.0 equiv; 6.47 mmol). Mesyl chloride (0.25 mL; 2.0 equiv; 3.23 mmol) was added dropwise and the reaction was left to warm to room temperature overnight. Then, sat. $NaHCO_3$ solution (15 mL) was added and the reaction mixture was transferred into separatory funnel. The organic layer was separated and the aqueous layer was washed with DCM two more times; organic layers were combined and dried using $Na_2SO_4$. Then, the salt was filtered off, and the solvent was evaporated. The product was purified using column chromatography, eluent system Hex/MTBE/acetone 4:1:1, product $R_f \approx 0.4$). The product *S*-**6** (260 mg; 75%) was obtained as a clear oil.

**$^1$H NMR** (400 MHz, $CDCl_3$, ppm) δ 6.41 (dd, *J* = 16.1, 4.1 Hz, 1H), 6.32 (dd, *J* = 16.1, 1.0 Hz, 1H), 4.90 (dd, *J* = 4.1, 1.0 Hz, 1H), 4.32 – 4.23 (m, 1H), 4.02 – 3.92 (m, 2H), 3.329 and 3.327 (both s, total 6H), 1.82 – 1.71 (m, 1H), 0.97 (d, *J* = 6.7 Hz, 3H), 0.88 (d, *J* = 6.8 Hz, 3H).

**$^{13}$C{$^1$H} NMR** (101 MHz, $CDCl_3$, ppm) δ 162.2, 137.7, 121.4, 101.4, 72.8, 70.1, 53.0, 53.0, 32.9, 19.0, 18.4.

**HRMS** (ESI) m/z: $[M+H]^+$ Calcd for $C_{11}H_{20}NO_3$ 214.1438; Found 214.1454.

*S*-**6**: $\alpha_D^{20} - 99.4$ ($c$ 1.01; $CHCl_3$).

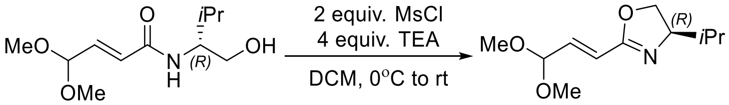


Synthesis of *R*-**6** was performed using analogous procedure as for *S*-**6**. Substrate *trans-R*-**5** (207 mg; 1.0 equiv; 0.90 mmol), TEA (0.75 mL; 6.0 equiv; 5.37 mmol) and mesyl chloride (0.21 mL; 3.0 equiv; 2.68 mmol) were used. Product *R*-**6** (133 mg; 70%) was obtained as a colorless oil.

**$^1$H NMR** and **$^{13}$C NMR** spectra for *R*-**6** matches that of *S*-**6**.

*R*-**6**: $\alpha_D^{20} + 96.9$ ($c$ 1.00; $CHCl_3$).

**Synthesis of oxazolo[3,2-*c*]pyrimidine 7**

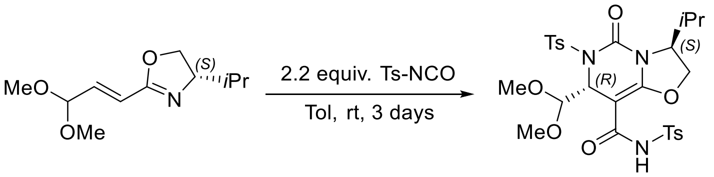


Substrate *S*-**6** (114 mg; 1.0 equiv; 0.53 mmol) was dissolved in dry toluene (5 mL). Tosyl isocyanate (0.18 mL; 2.2 equiv; 1.18 mmol) was added dropwise and the reaction mixture was left to stir at room temperature. Reaction progress was monitored with UPLC–MS. After 3 days, all starting material was consumed. The solvent was evaporated and the crude product was purified, using column chromatography, eluent system petroleum ether/MTBE/acetone 2:1:1, product $R_f \approx 0.3$), followed by flash chromatography on reversed phase, using a C18 silica gel column. Eluent system $H_2O$/MeCN, gradient 20% to 80% MeCN. Product **7** (280 mg; 86%) was obtained as an amorphous white solid. The product was obtained as a single diastereomer.

**$^1$H NMR** (400 MHz, $CD_3CN$, ppm) δ 8.93 (s, 1H), 7.95 – 7.88 (m, 2H), 7.88 – 7.79 (m, 2H), 7.45 – 7.37 (m, 2H), 7.38 – 7.29 (m, 2H), 5.35 (d, *J* = 2.4 Hz, 1H), 4.66 – 4.52 (m, 2H), 4.32 – 4.25 (m, 1H), 4.02 (d, *J* = 2.4 Hz, 1H), 3.26 (s, 3H), 3.23 (s, 3H), 2.44 (s, 3H), 2.40 (s, 3H), 2.35 (ddt, *J* = 10.4, 7.0, 3.4 Hz, 1H), 0.80 (d, *J* = 7.2 Hz, 3H), 0.45 (d, *J* = 6.9 Hz, 3H).

**$^{13}$C{$^1$H} NMR** (101 MHz, $CD_3CN$, ppm) δ 161.5, 157.4, 148.4, 146.3, 145.9, 137.7, 137.1, 130.5, 130.1, 129.9, 129.0, 118.3, 107.0, 78.1, 72.2, 61.3, 58.0, 56.0, 55.2, 27.1, 21.7, 21.6, 18.3, 13.9.

**HRMS** (ESI) m/z: $[M+H]^+$ Calcd for $C_{27}H_{34}N_3O_9S_2$ 608.1730; Found 608.1755.

**7**: $\alpha_D^{20} + 158.5\ (c\ 1.01; \mathrm{CHCl_3})$.

## Chiral HPLC analysis of *rac*-6

*Rac*-**6** was synthesized using the same methodology as for *S*-**6** and *R*-**6**. DL-Valinol (CAS: 16369-05-4) was used as starting material.

HPLC (Chiralpak IG, isopropanol/*n*-hexane 10:90, flow rate = 1.0 mL/min, λ = 210 nm) $t_R$ = 8.43 min (isomer A), 9.90 min (isomer B)

### SAMPLE INFORMATION

| | | | |
|---|---|---|---|
| Sample Name: | 616-Ch-390-MD-S659 | | |
| Sample Type: | Unknown | Sample Set Name: | 07072021_Ch390 |
| Vial: | 26 | Acq. Method Set: | Iz_210_254_F1_100B |
| Injection #: | 1 | Processing Method | CH_390_2 |
| Injection Volume: | 10.00 ul | Channel Name: | W2489 ChA |
| Run Time: | 20.0 Minutes | Proc. Chnl. Descr.: | W2489 ChA 210nm |
| Date Acquired: | 7/7/2021 2:16:50 PM EET | Acquired By: | System |
| Date Processed: | 7/8/2021 8:56:03 AM EET | | |

Chiralpak IC-2 (4.6x250 mm)
10%IPA/HEX; F=1 mL/min; T=25oC

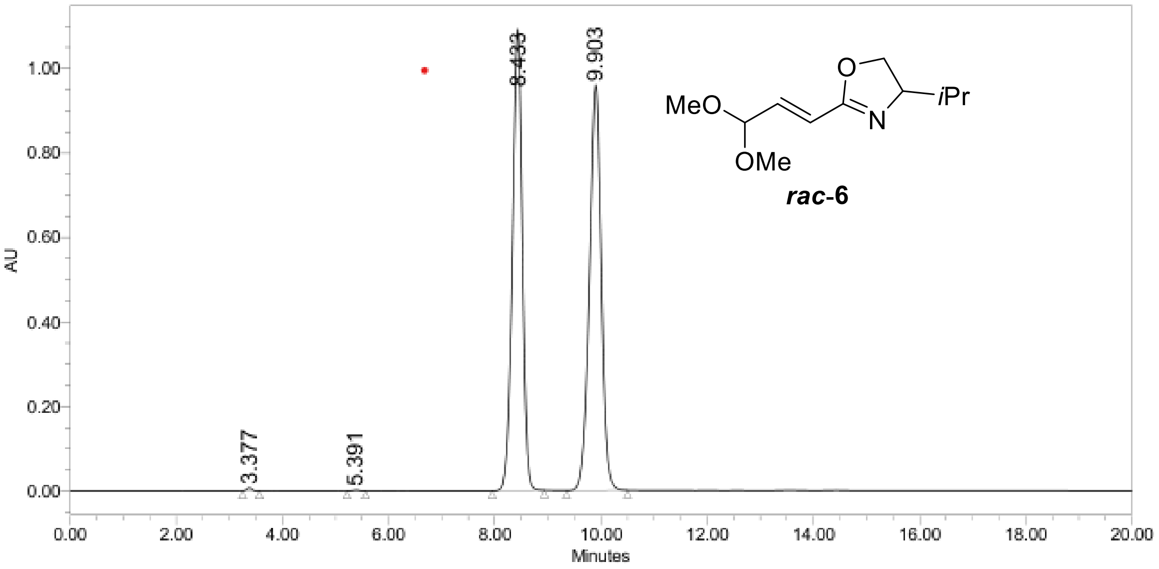


1 C~ 1mg/ml in 10%IPA_Hexane

| | RT | Area | % Area | Height | EP Plate Count | Resolution | Selectivity | Width @ 50% | K Prime |
|---|---|---|---|---|---|---|---|---|---|
| 1 | 3.377 | 43654 | 0.15 | 6581 | 5842 | | | 0.104 | 0.036 |
| 2 | 5.391 | 15250 | 0.05 | 1823 | 9226 | 10.064 | 18.161 | 0.132 | 0.654 |
| 3 | 8.433 | 14070037 | 48.57 | 1090953 | 10200 | 10.922 | 2.427 | 0.197 | 1.587 |
| 4 | 9.903 | 14838525 | 51.22 | 960047 | 9964 | 4.034 | 1.284 | 0.234 | 2.038 |

## Chiral HPLC analysis of *S*-6

HPLC (Chiralpak IG, isopropanol/*n*-hexane 10:90, flow rate = 1.0 mL/min, $\lambda$ = 210 nm) $t_R$ =8.34 min (isomer A), 9.88 min (isomer B)

### SAMPLE INFORMATION

| | | | |
|---|---|---|---|
| Sample Name: | 536-Ch-390-MD-S642 | | |
| Sample Type: | Unknown | Sample Set Name: | 03062021_Ch390 |
| Vial: | 53 | Acq. Method Set: | Iz_210_254_F1_100B |
| Injection #: | 1 | Processing Method | CH_390_2 |
| Injection Volume: | 10.00 ul | Channel Name: | W2489 ChA |
| Run Time: | 20.0 Minutes | Proc. Chnl. Descr.: | W2489 ChA 210nm |
| Date Acquired: | 6/3/2021 1:27:24 PM EET | Acquired By: | System |
| Date Processed: | 6/3/2021 2:18:50 PM EET | | |

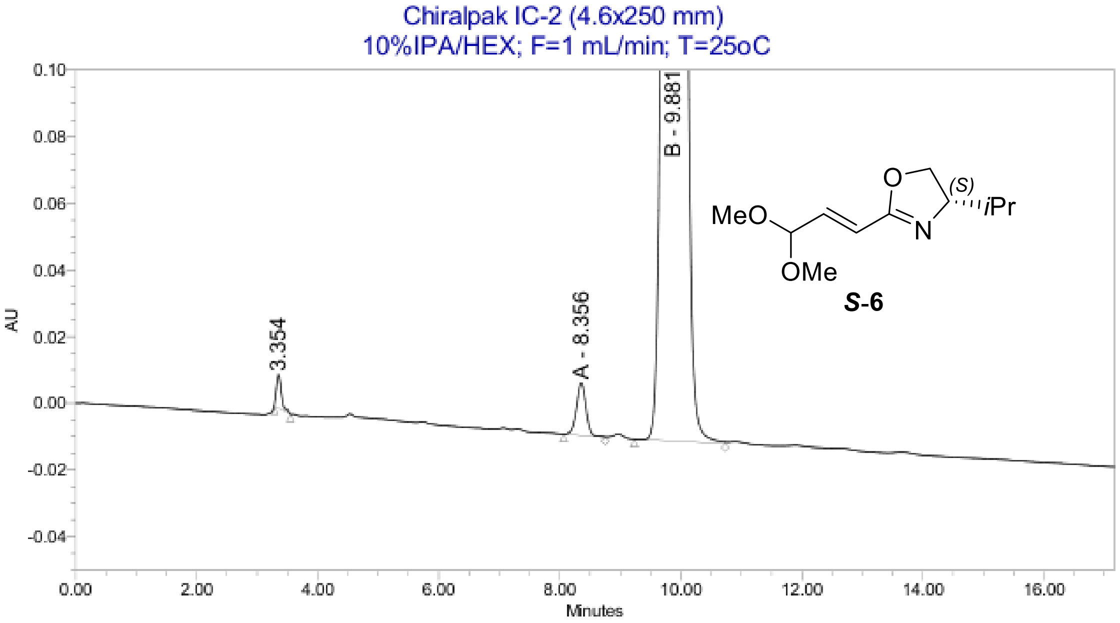


1 C~ 1 mg/ml in 10%IPA_Hexane

| | Peak Name | RT | Area | % Area | Height | EP Plate Count | Resolution | Selectivity | Width @ 50% | K Prime |
|---|---|---|---|---|---|---|---|---|---|---|
| 1 | | 3.354 | 56464 | 0.19 | 10082 | 7965 | | | 0.088 | 0.029 |
| 2 | A | 8.356 | 171522 | 0.58 | 15868 | 14009 | 23.177 | 53.934 | 0.166 | 1.563 |
| 3 | B | 9.881 | 29282010 | 99.23 | 2069115 | 11602 | 4.709 | 1.299 | 0.216 | 2.031 |

## Chiral HPLC analysis of *R*-6

HPLC (Chiralpak IG, isopropanol/*n*-hexane 10:90, flow rate = 1.0 mL/min, λ = 210 nm) $t_R$ = 8.23 min (isomer A), 9.73 min (isomer B)

### SAMPLE INFORMATION

| | | | |
|---|---|---|---|
| Sample Name: | 464_Ch-390-MD-S620 | | |
| Sample Type: | Unknown | Sample Set Name: | 17_05_2021 |
| Vial: | 18 | Acq. Method Set: | Iz_210_254_F1_90D_10B_25oC |
| Injection #: | 1 | Processing Method | Chiralpak IC 1 |
| Injection Volume: | 10.00 ul | Channel Name: | W2489 ChB |
| Run Time: | 15.0 Minutes | Proc. Chnl. Descr.: | W2489 ChB 210nm |
| Date Acquired: | 5/17/2021 11:22:56 AM EET | Acquired By: | System |
| Date Processed: | 5/17/2021 12:22:21 PM EET | | |

Chiralpak IC-1 (4.6x250 mm)
Iz.10% IPA/90%HEX; F=1 mL/min; T=25oC

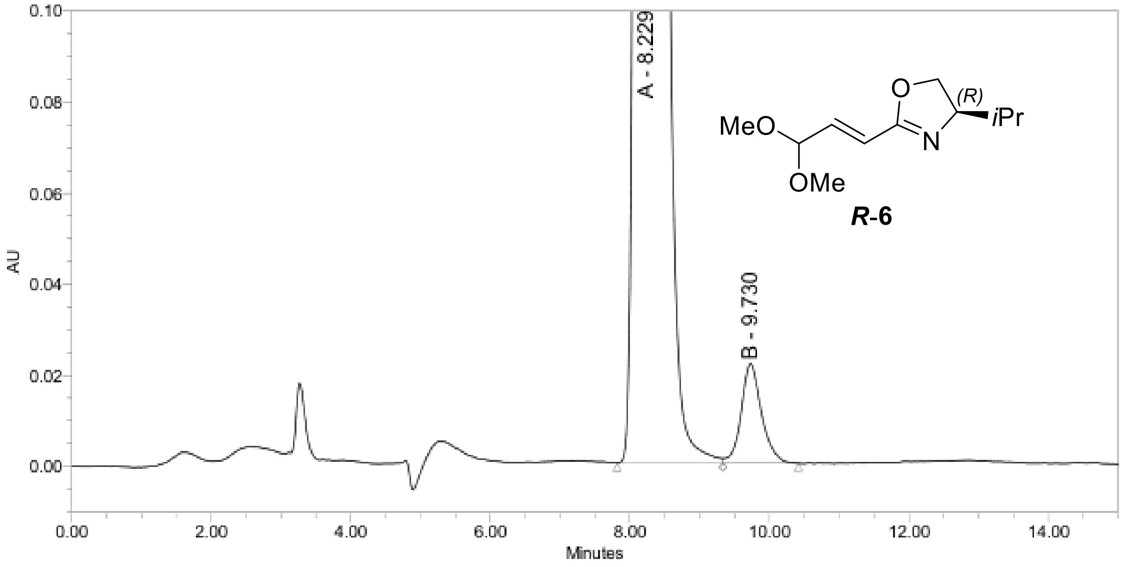


***R*-6**

c=1 mg/ml 35%EtOH/HEX

| | Peak Name | RT | Area | % Area | Height | EP Plate Count | Resolution | Selectivity | Width @ 50% | K Prime |
|---|---|---|---|---|---|---|---|---|---|---|
| 1 | A | 8.229 | 27588615 | 98.42 | 1703216 | 6464 | | | 0.241 | 1.612 |
| 2 | B | 9.730 | 442395 | 1.58 | 21871 | 6057 | 3.309 | 1.295 | 0.294 | 2.089 |

**Copies of NMR spectra**

**Figure S1**. $^{1}$H NMR of compound *S*-**S1** ($CDCl_3$, 300 MHz)

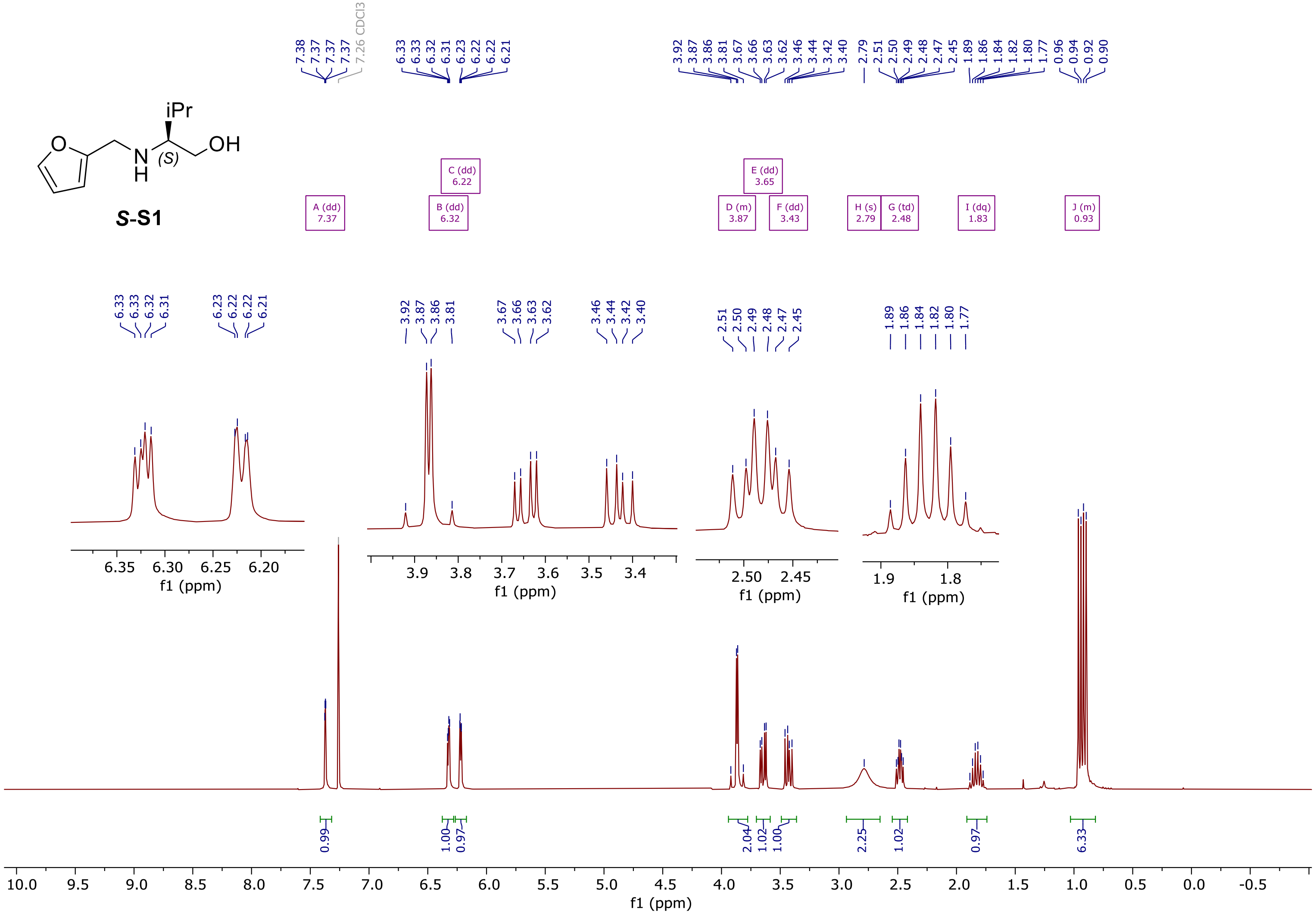

**Figure S2**. $^{1}$H NMR of compound *S*-**2d** (DMSO-$d_6$, 400 MHz)

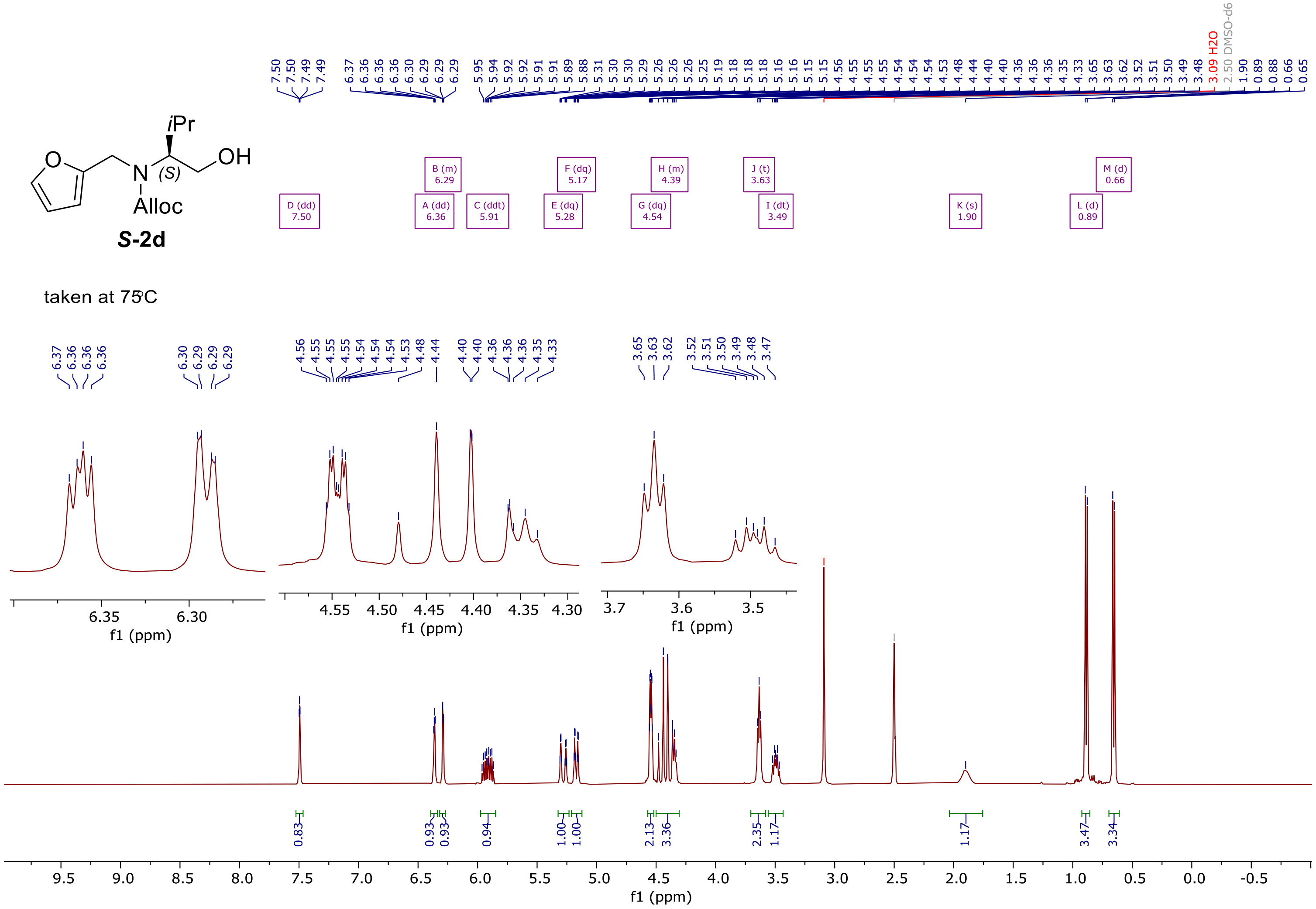

**Figure S3**. $^{13}C\{^{1}H\}$ NMR of compound *S*-**2d** (DMSO-$d_6$, 101 MHz)

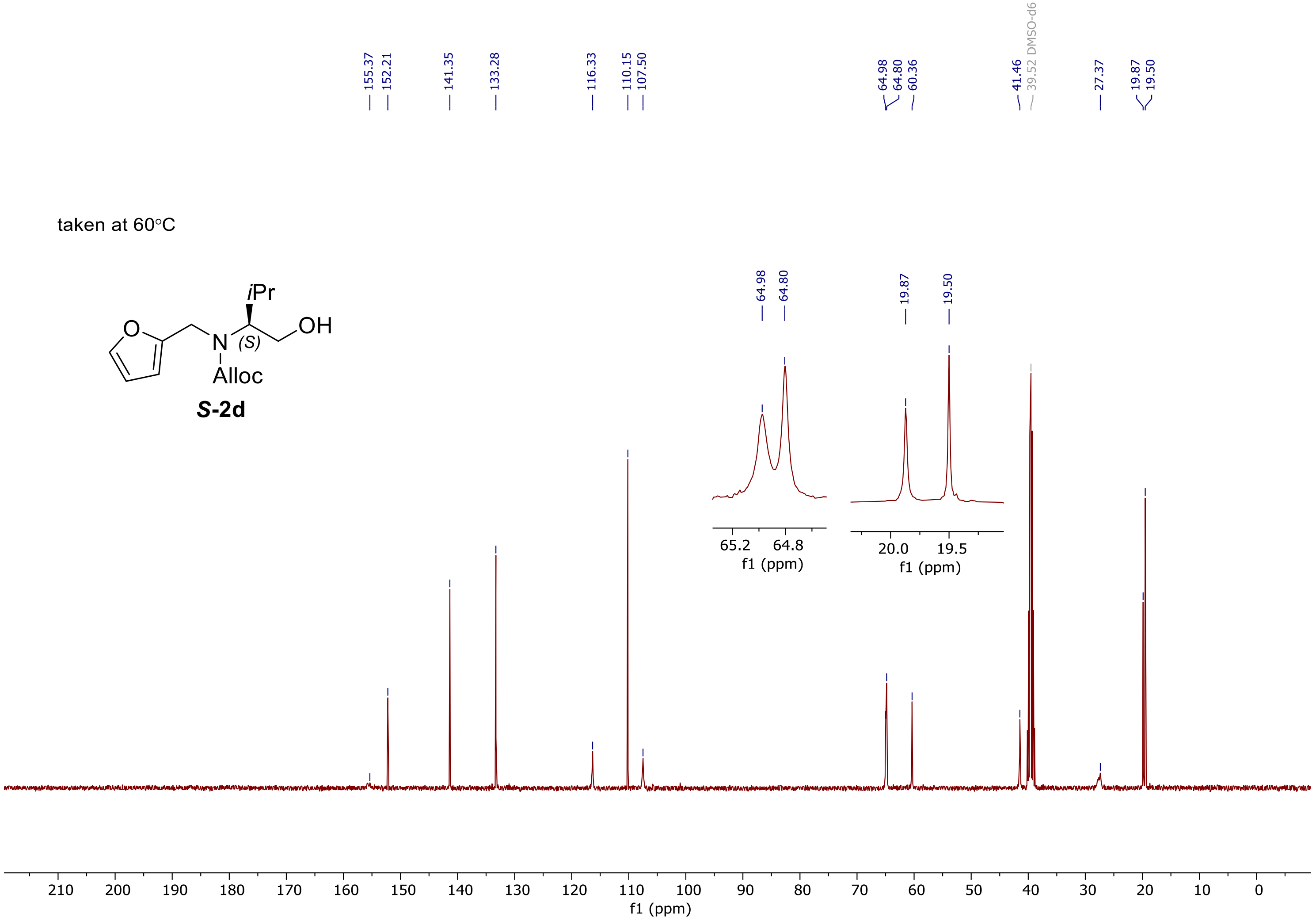

**Figure S4**. $^{1}$H NMR of compound *S*-**3d** ($CD_3CN$, 400 MHz)

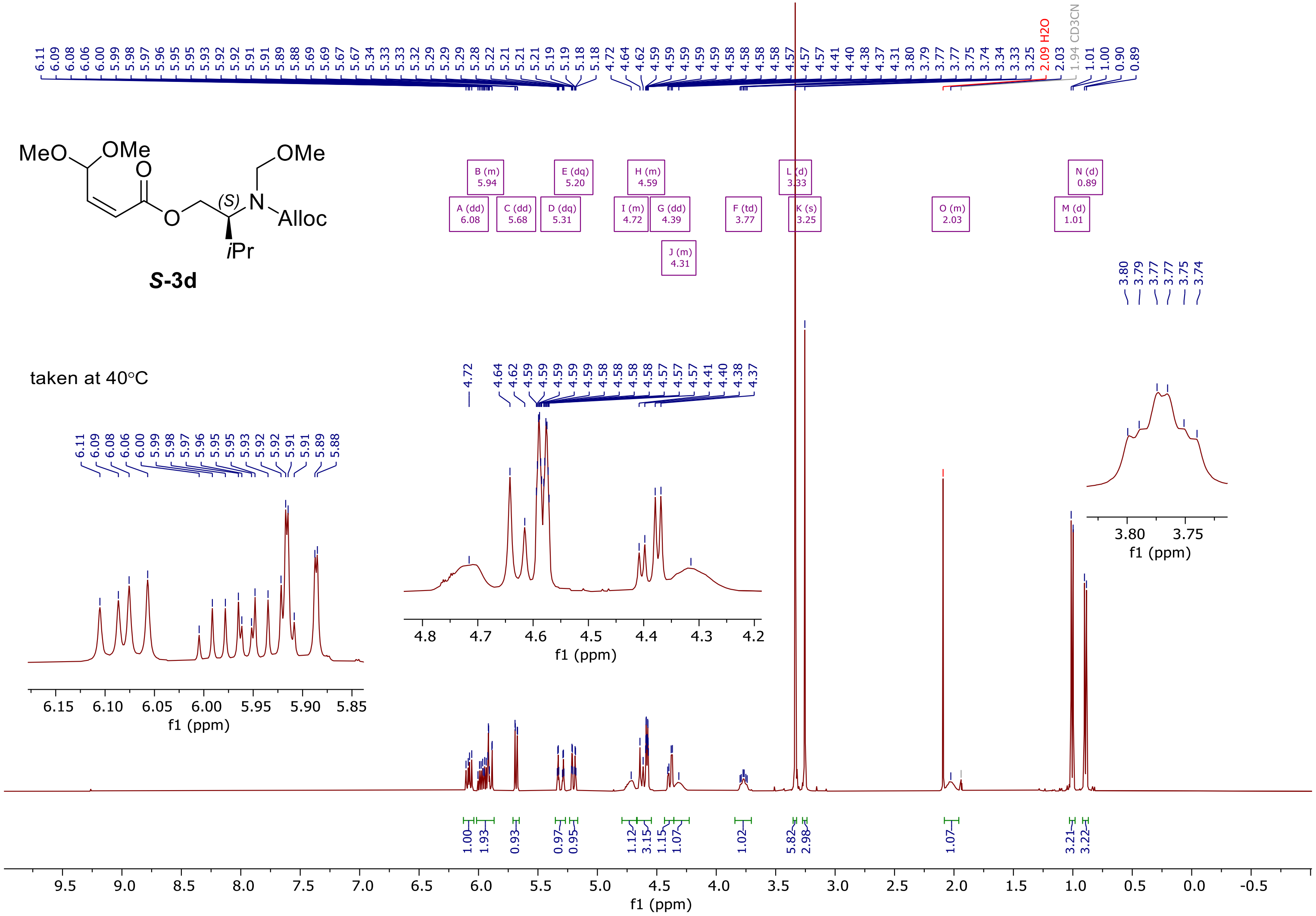

**Figure S5**. $^{13}C\{^{1}H\}$ NMR of compound *S*-**3d** ($CD_3CN$, 101 MHz).

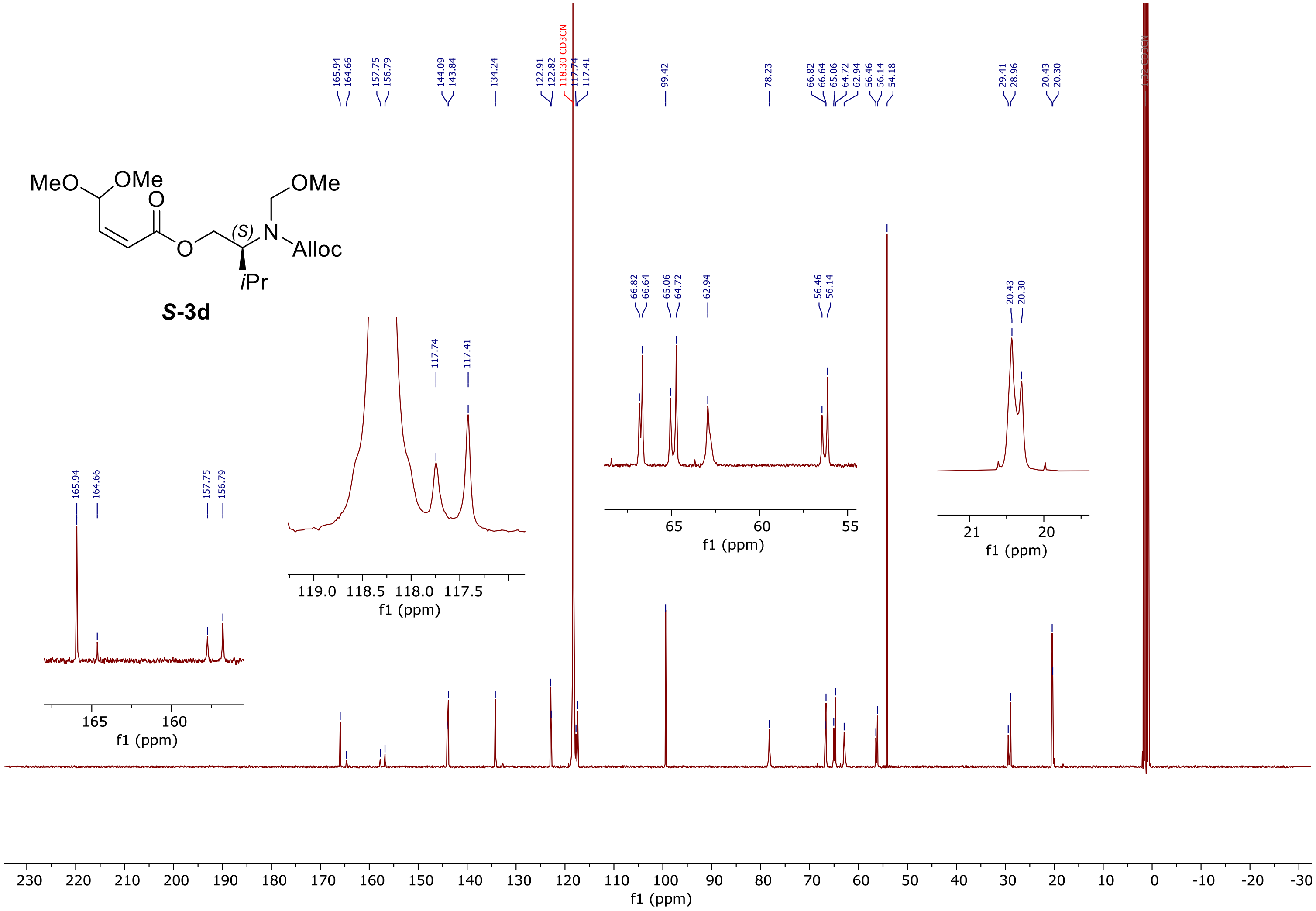

**Figure S6**. $^{1}$H NMR of compound *trans*-*S*-**5** ($CDCl_3$, 400 MHz)

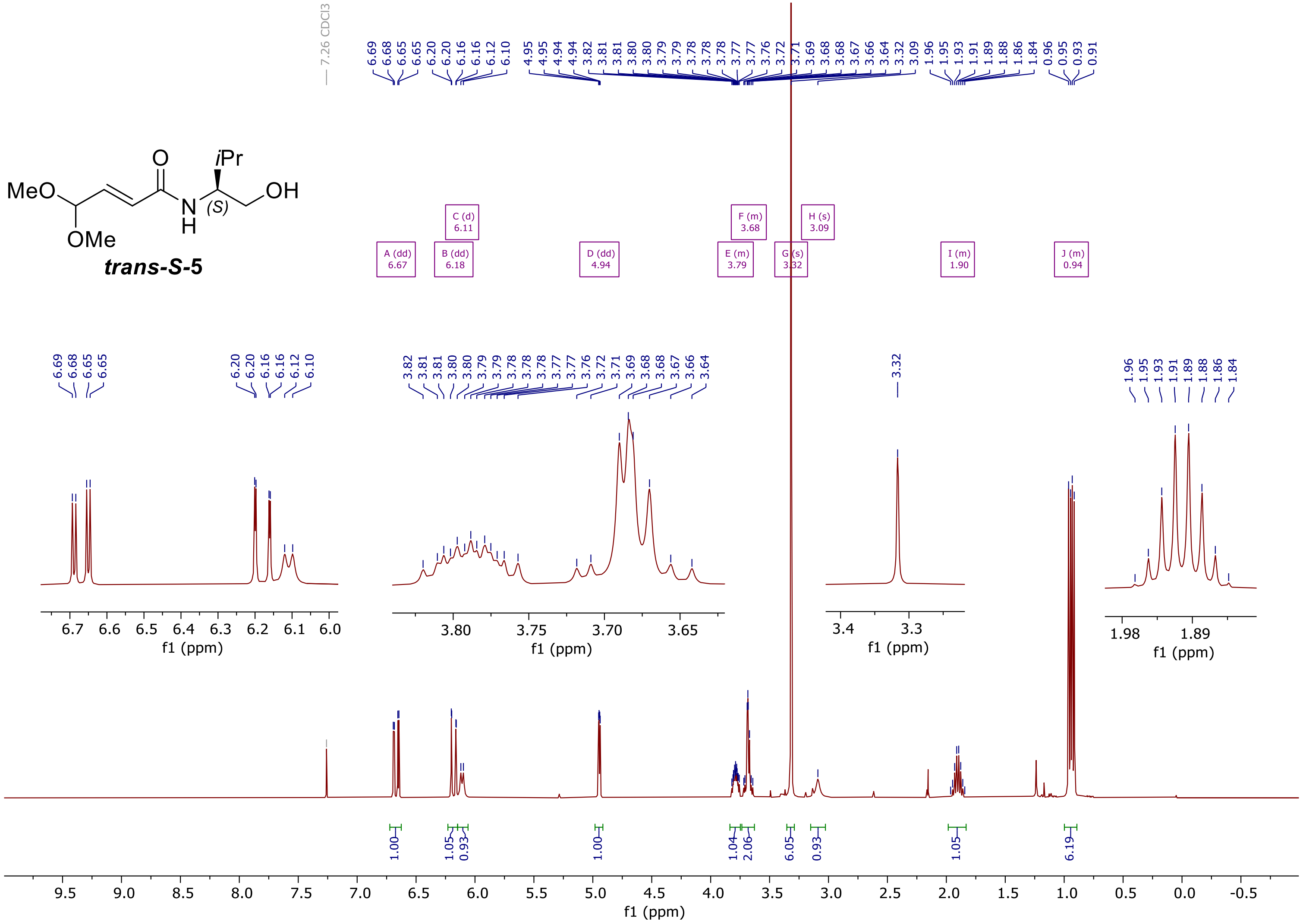

**Figure S7**. $^{13}C\{^1H\}$ NMR of compound *trans*-*S*-**5** ($CDCl_3$, 101 MHz).

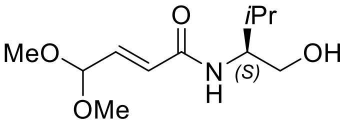


*trans-S-5*

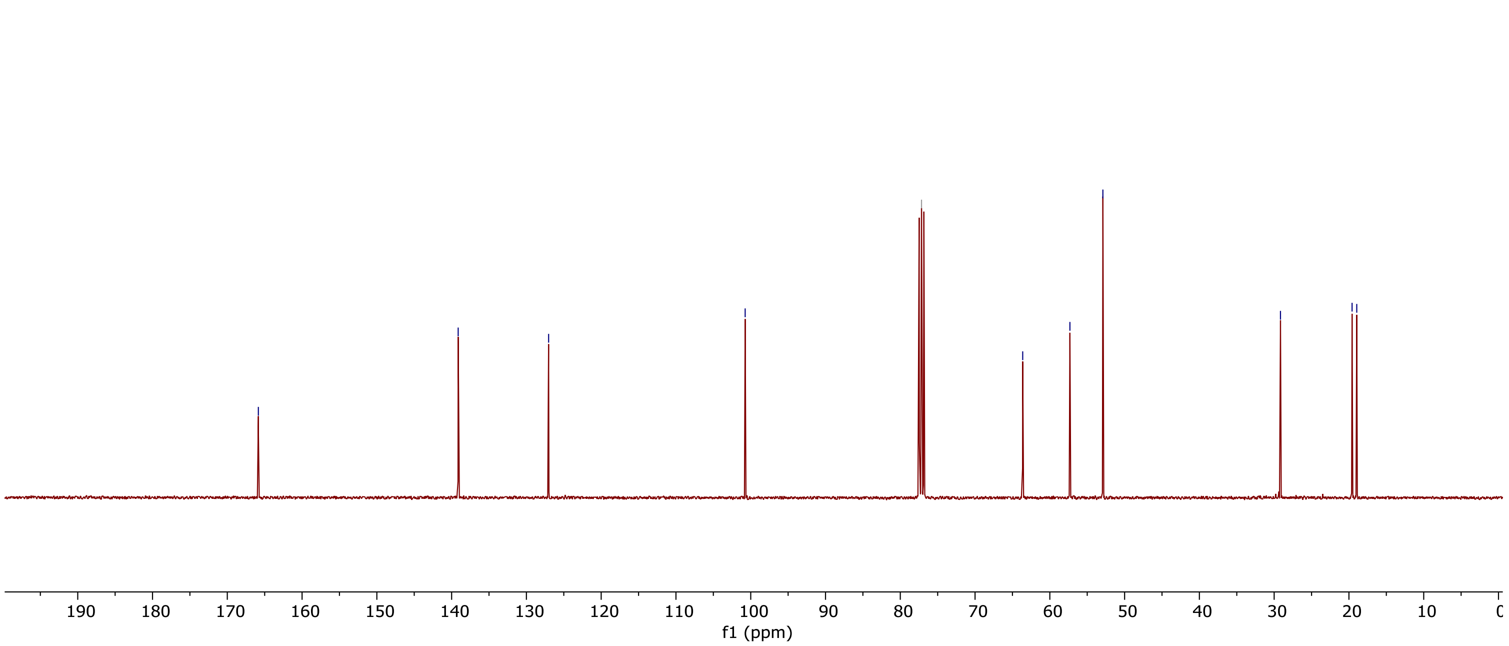

**Figure S8**. $^{1}$H NMR of compound *cis*-*S*-**5** ($CDCl_3$, 400 MHz).

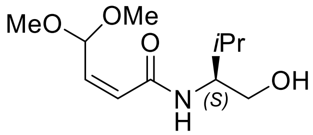


***cis-S-5***

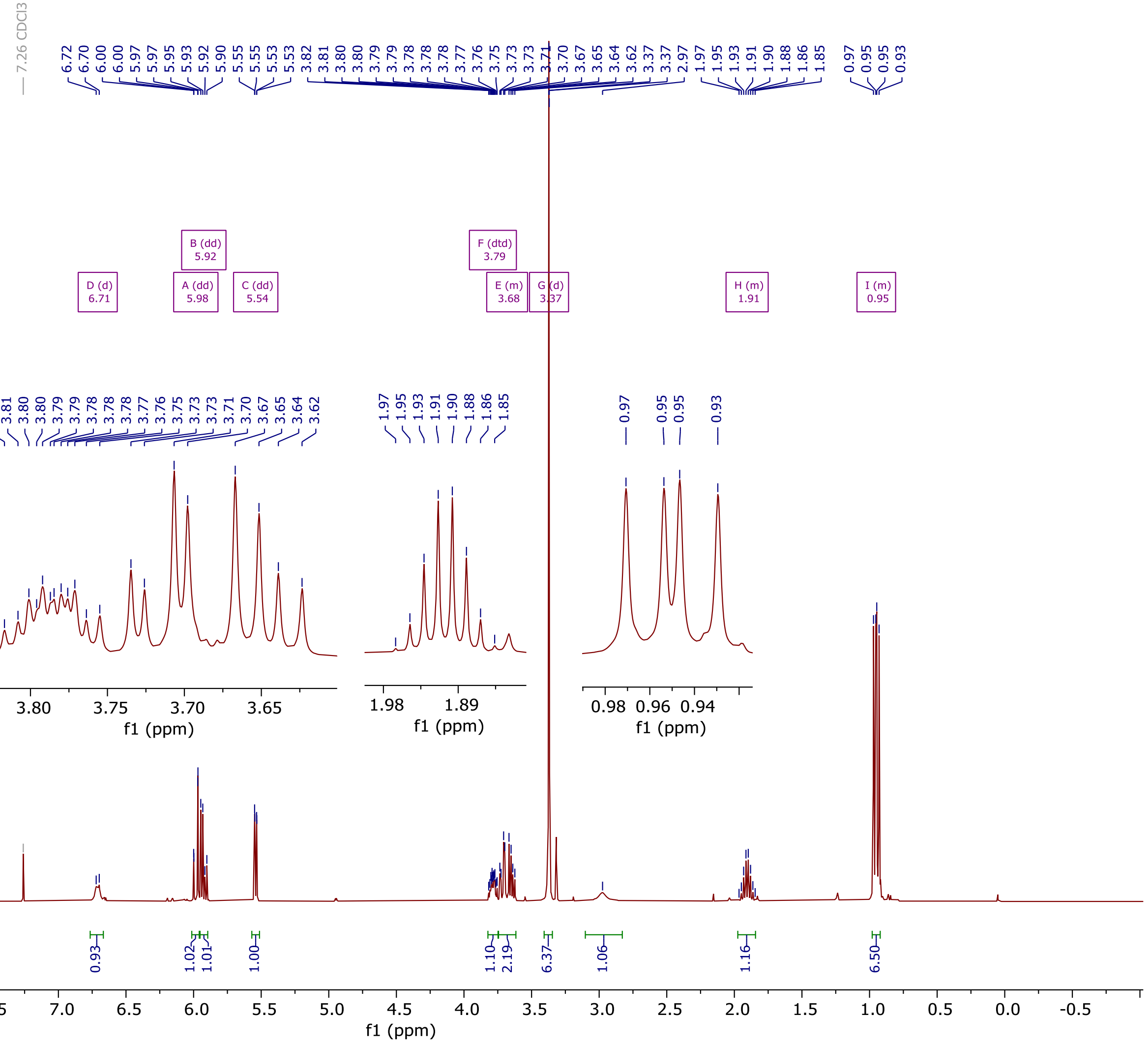

**Figure S9**. $^{13}C\{^{1}H\}$ NMR of compound *cis*-*S*-**5** ($CDCl_3$, 101 MHz).

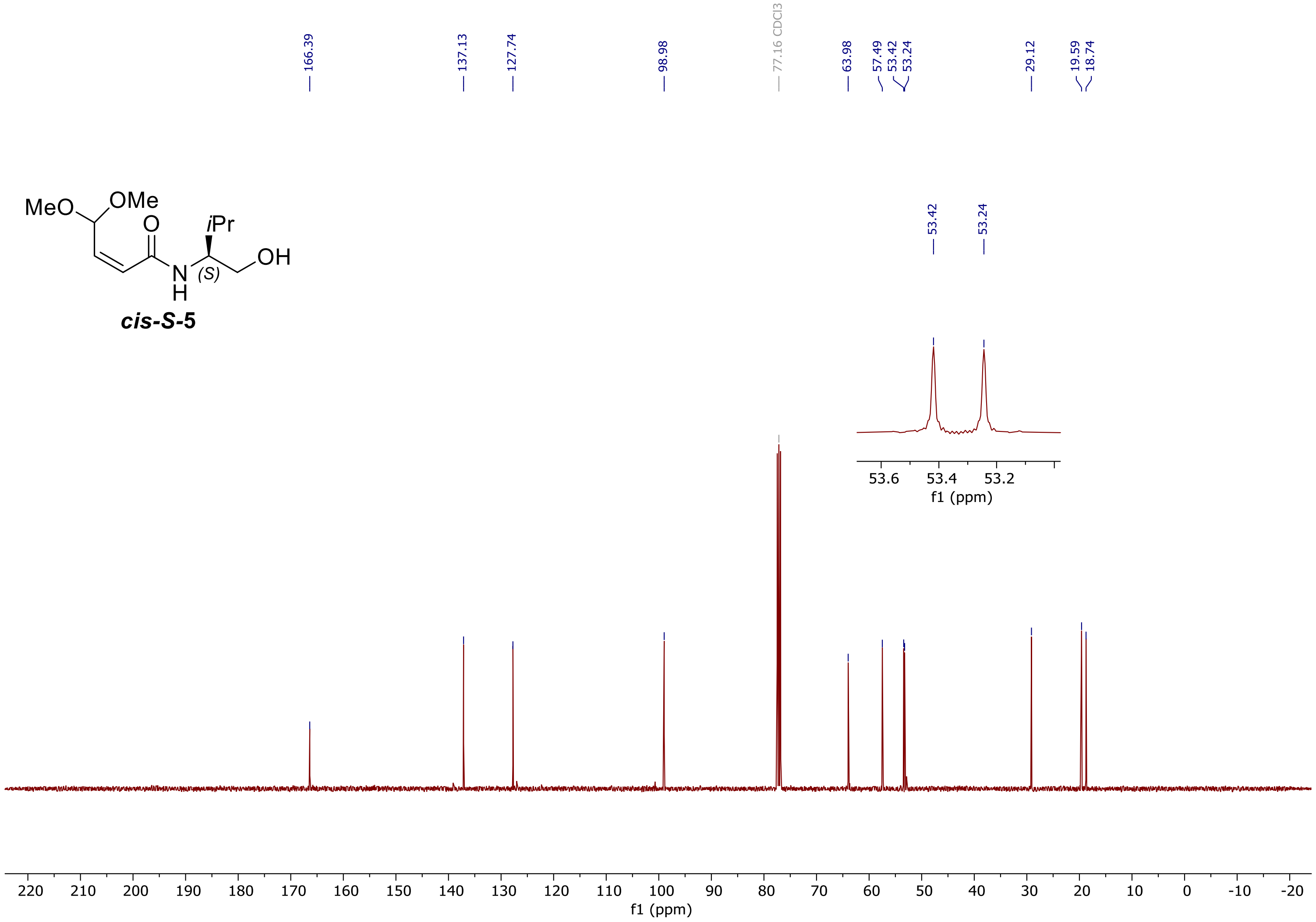

**Figure S10**. $^{1}$H NMR of compound *S*-**6** ($CDCl_3$, 400 MHz)

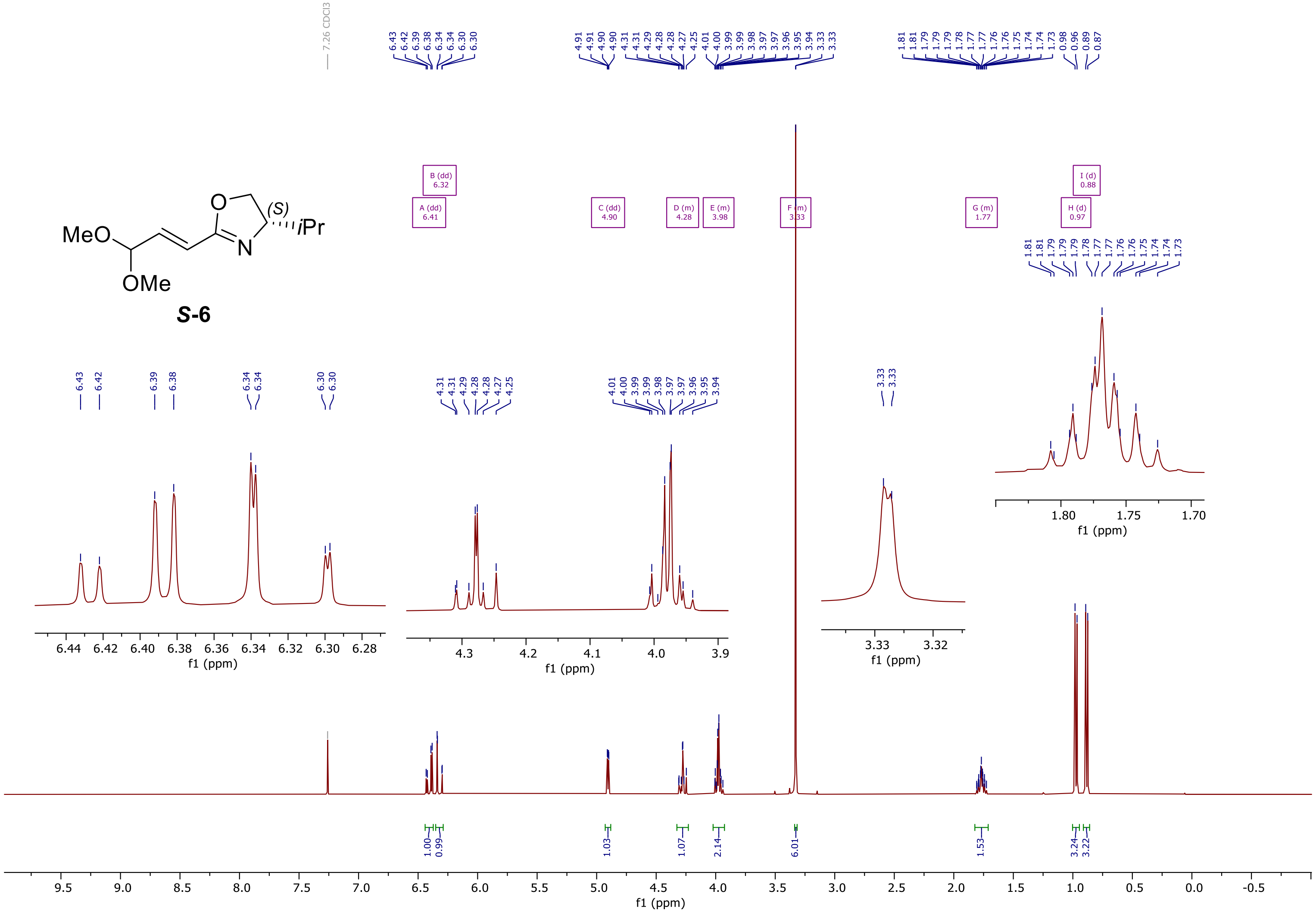

**Figure S11**. $^{13}C\{^{1}H\}$ NMR of compound *S*-**6** ($CDCl_3$, 101 MHz).

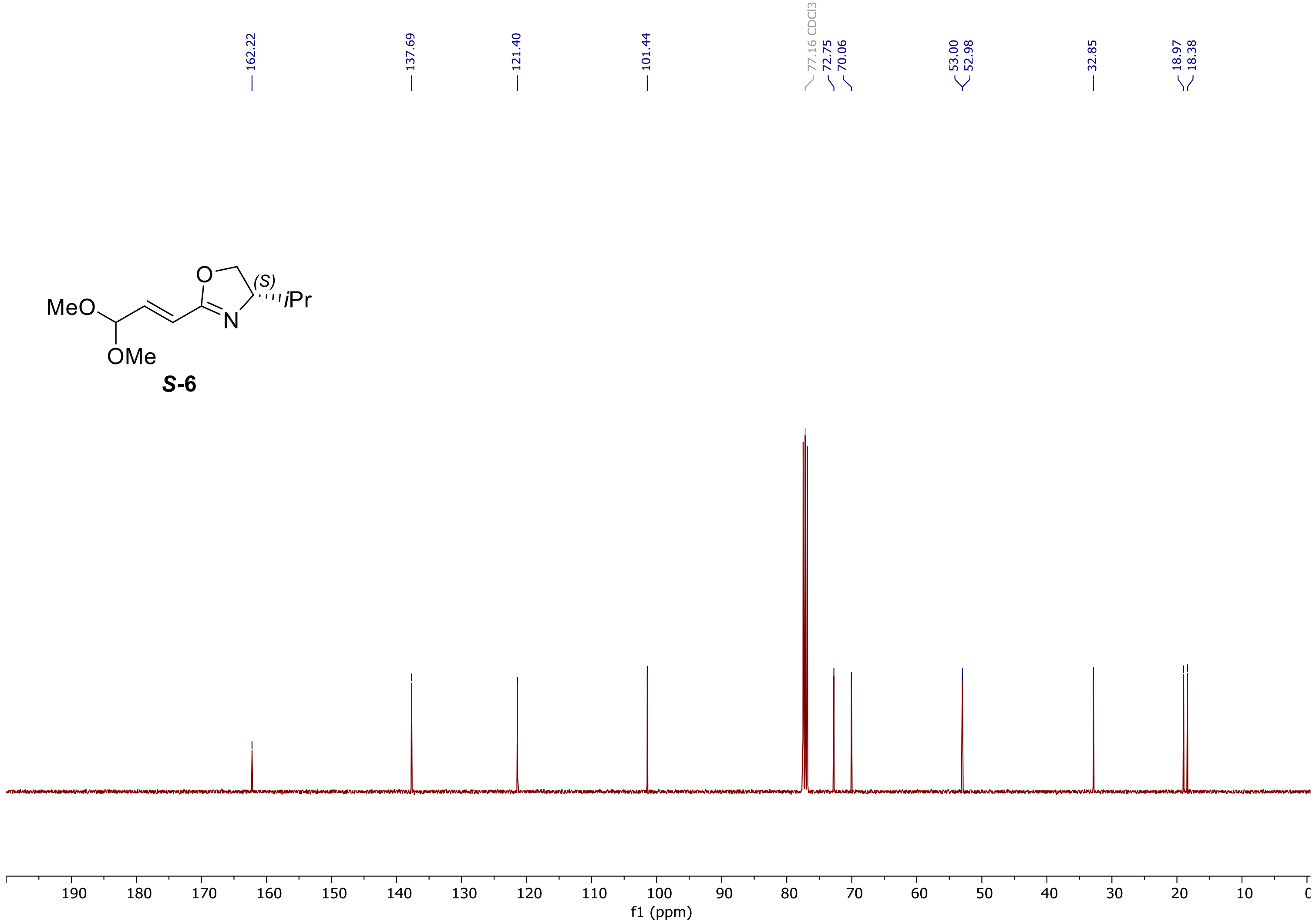

**Figure S12**. $^{1}$H NMR of compound **7** ($CD_3CN$, 400 MHz).

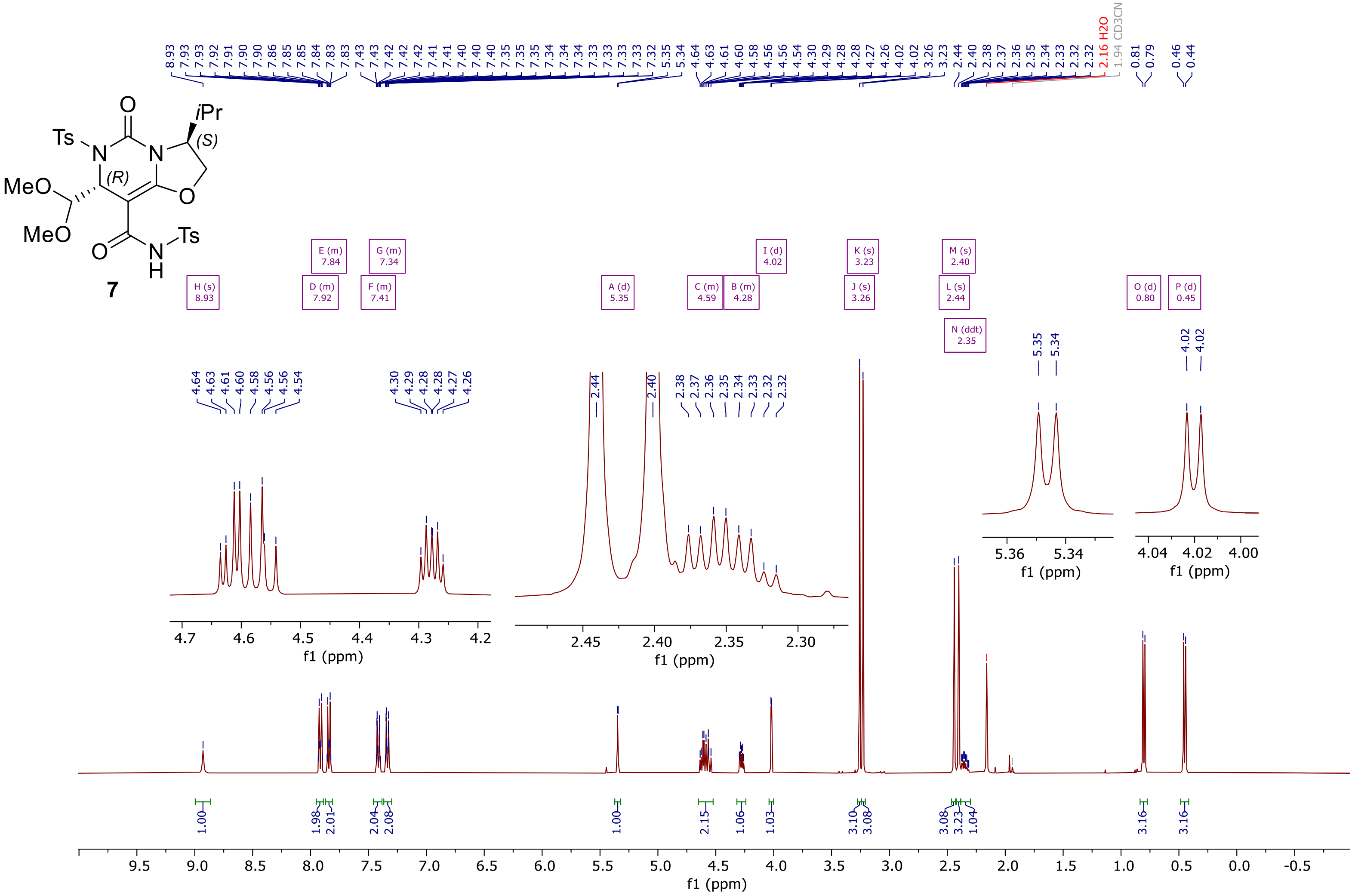

**Figure S13**. $^{13}C\{^1H\}$ NMR of compound **7** ($CD_3CN$, 101 MHz).

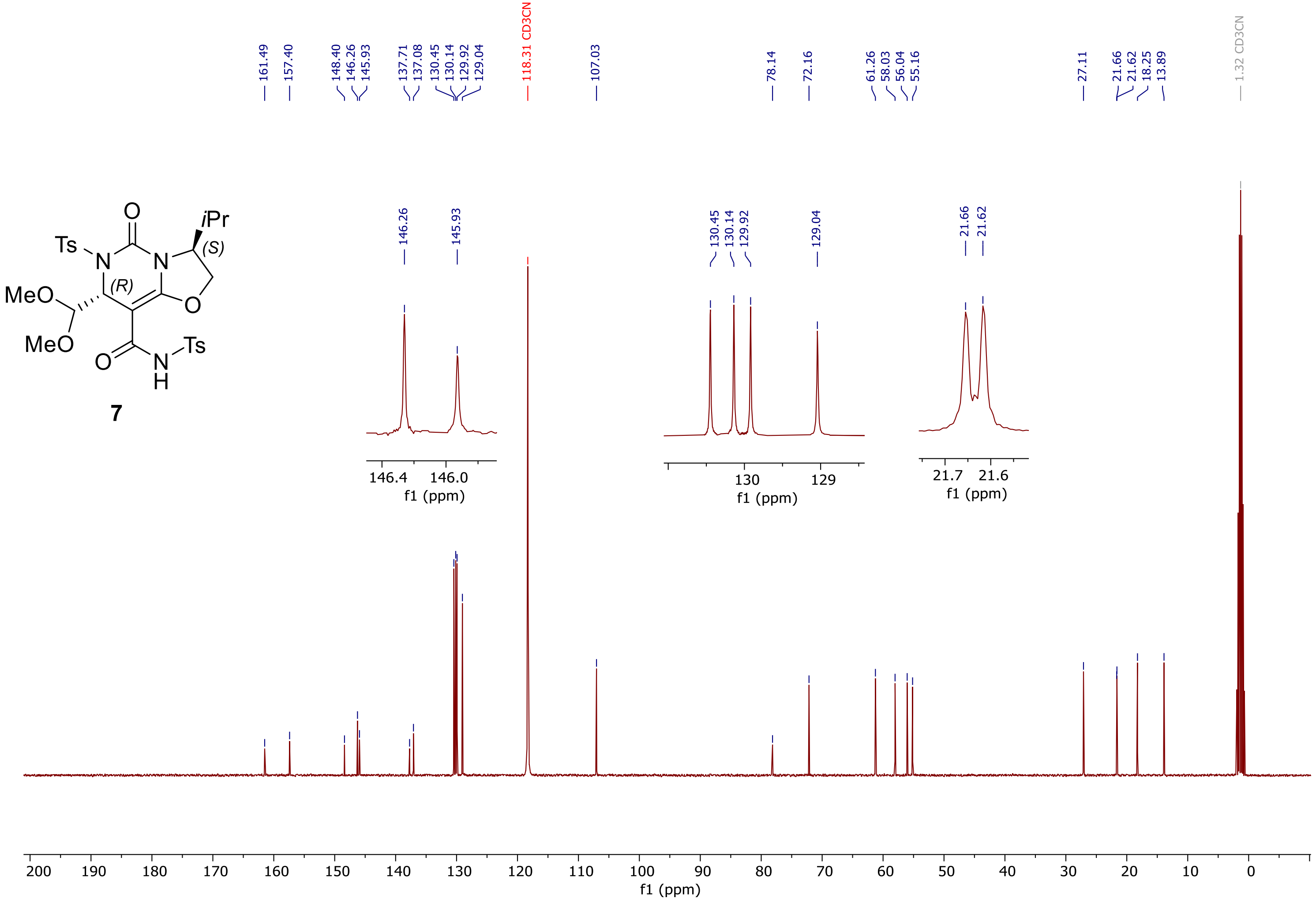

Supplement: File 1 — Experimental procedures, characterization data and copies of NMR spectra. [file Beilstein_J_Org_Chem-21-1737-s001.pdf]
